# Divergent Approach for *Tris*-Heteroleptic Cyclometalated Iridium Complexes Using Triisopropylsilylethynyl-Substituted Synthons

**DOI:** 10.1021/acs.organomet.2c00292

**Published:** 2022-08-19

**Authors:** Robert M. Edkins, Yu-Ting Hsu, Mark A. Fox, Dmitry Yufit, Andrew Beeby, Ross J. Davidson

**Affiliations:** † Department of Chemistry, University of Durham, South Road, England, Durham DH1 3LE, U.K.; ‡ WestCHEM Department of Pure and Applied Chemistry, University of Strathclyde, 295 Cathedral Street, Glasgow G1 1XL, U.K.

## Abstract

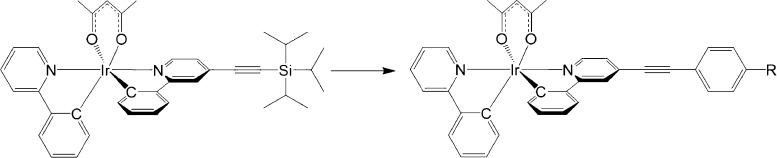

*Bis*-heteroleptic cyclometalated iridium complexes of the form Ir(L^a^)_2_(acac), where L^a^ is a substituted 2-phenylpyridine derivative and acac is an acetylacetonato ligand, are a useful class of luminescent organometallic complexes for a range of applications. Related *tris*-heteroleptic complexes of the form Ir(L^a^)(L^b^)(acac) offer the potential advantage of greater functionality through the use of two different cyclometalated ligands but are, in general, more difficult to obtain. We report the synthesis of divergent *bis*- and *tris*-heteroleptic triisopropylsilylethynyl-substituted intermediate complexes that can be diversified using a “chemistry-on-the-complex” approach. We demonstrate the methodology through one-pot deprotection and Sonogashira cross-coupling of the intermediate complexes with *para*-R-aryliodides (R = H, SMe, and CN). The photophysical and electrochemical behaviors of the resultant *bis*- and *tris*-heteroleptic complexes are compared, and it is shown that the *tris*-heteroleptic complexes exhibit subtly different emission and redox properties to the *bis*-heteroleptic complexes, such as further red-shifted emission maxima and lower extinction coefficients, which can be attributed to the reduced symmetry. It is demonstrated, supported by DFT and time-dependent DFT calculations, that the charge-transfer character of the emission can be altered via variation of the terminal substituent; the introduction of an electron-withdrawing cyano group in the terminal position leads to a significant red shift, while the introduction of an SMe group can substantially increase the emission quantum yield. Most notably, this convenient synthetic approach reduces the need to perform the often challenging isolation of *tris*-heteroleptic complexes to a single divergent intermediate, which will simplify access to families of complexes of the form Ir(L^a^)(L^b^)(acac).

Iridium complexes containing a *bis*-heteroleptic arrangement Ir(N^C)_2_(A) (where N^C is a 2-phenylpyridine-based ligand and A = bidentate ancillary ligand) were first synthesized by Lamansky^1^ in 2001 and have since become extensively used for applications such as organic light-emitting diodes (OLEDs),^2^ biological imaging,^3^ and, more recently, photocatalysts.^4^ Their ubiquitous use can be attributed to their predictable structure–property relationships and controllable electronic structure, that is, the LUMO is predominantly localized to the pyridyl group, while the HOMO is localized to the iridium and phenyl groups, and, as such, any modifications at these positions can tune the emission color.^5−9^ Additionally, the emission lifetimes are typically in the microsecond range, with photoluminescent-quantum yields (PLQYs, Φ), in general, higher than those of other similar organometallic systems.

Despite *bis*-heteroleptic iridium complexes being well-established, there remain relatively few examples of *tris*-heteroleptic iridium complexes.^10^ It was not until we developed the approach of reacting two different 2-phenylpyridine-based ligands (L^a^ and L^b^) with IrCl_3_·3H_2_O to form a mixture of iridium chloro-bridged dimers followed by reaction with acetylacetone to give a statistical mixture of Ir(L^a^)_2_(acac), Ir(L^a^)(L^b^)(acac), and Ir(L^b^)_2_(acac) species that Ir(L^a^)(L^b^)(acac) complexes could be separated by chromatography.^10−12^ Following this, new approaches have been developed that initially use the degradation of a *tris*-cyclometalated to a mixed ligand iridium-chloro dimer, which was coordinated to an ancillary ligand to form a *tris*-heteroleptic complex.^13−15^ Adamovich et al. recently reported an approach that used an aryl-*N*-heterocyclic carbene ligand to form an intermediate iridium complex that favored the formation of mixed ligand chloro dimers, avoiding statistical distributions of products.^16^

*Tris*-heteroleptic complexes have the benefit of combining properties originating from each individual ligand–metal combination into a single complex that can be additive, resulting in dual emissions, or synergistic, as in the case of a donor–acceptor-based complex that simultaneously enhanced both hole and electron transport of the complex to improve OLED emitter efficiency.^14,17−20^

However, in each of these examples, the synthesis was initiated by coordinating the complete ligand directly with iridium. This approach limits the scope for divergent synthesis and restricts the ligand design to contain only functionalities that will remain stable during the iridium coordination reaction. Recently, two groups established a possible solution for both of these issues. Bondreault et al. used an approach of reacting 2-(2′-bromophenyl)pyridine (Brppy) with [Ir(μ-Cl)(η^2^-C_8_H_14_)_2_]_2_, which, through a C–Br bond cleavage of one Brppy molecule, gave an Ir(Brppy)(ppy)(acac) complex. The bromo group was then coupled with various substrates by a Suzuki–Miyaura reaction to give a series of modified complexes.^21^ Hisamatsu et al., rather than starting with a *tris*-heteroleptic complex precursor, used an unsymmetric ancillary ligand to direct the selective iodination of the 2-phenylpyridine ligand, facilitating further modification of the complex.^18^ Building on this work, we report an additional approach for the divergent synthesis of *tris*-heteroleptic iridium complexes. Previously, we have demonstrated the utility of ethynyl-triisopropylsilylane (≡TIPS)-substituted *bis*-heteroleptic complexes to produce iridium complexes with high aspect ratios.^22^ In this report, we sought to combine the use of a ≡TIPS-substituted ligand with the statistical mixture approach to develop a synthon that could be used for divergent synthesis.

## Synthesis

**Figure 1:**
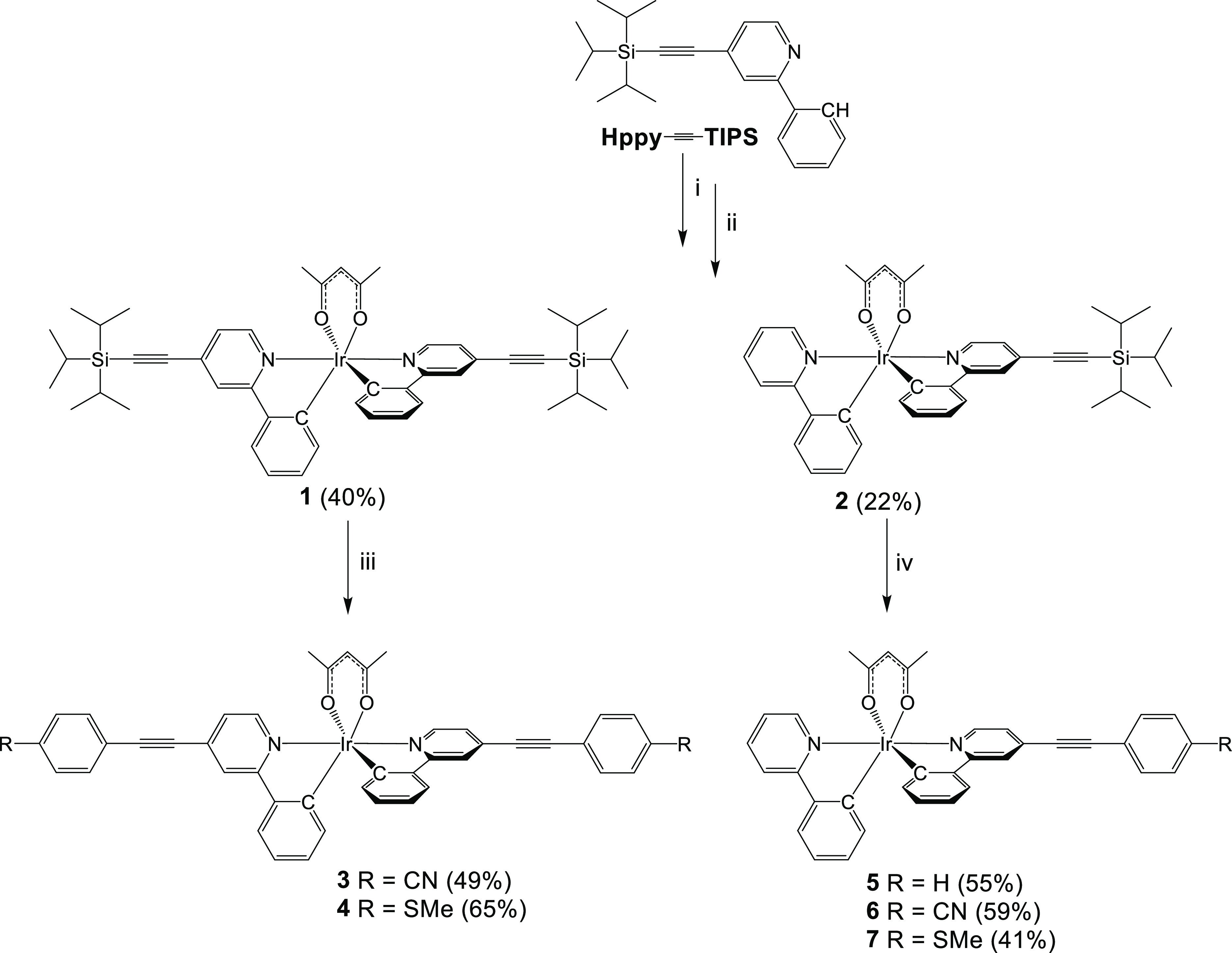
Synthetic scheme for complexes **1–7** where (i) 2.5 equiv IrCl_3_·3H_2_O, 2-ethoxyethanol, and 2.5 equiv Hppy; (ii) acetylacetone, K_2_CO_3_, and 2-ethoxyethanol; (iii) tetra-*n*-butylammonium fluoride (TBAF), 2 equiv I–Ar, Pd(PPh_3_)_4_, CuI, Et_3_N, and THF; and (iv) TBAF, 1 equiv I–Ar, Pd(PPh_3_)_4_, CuI, Et_3_N, and THF.

A mixture of 2-phenyl-4-((triisopropylsilyl)ethynyl)pyridine (Hppy≡TIPS)^22^ and 2-phenylpyridine (Hppy) (1:2.5 equiv) was reacted with IrCl_3_·3H_2_O (2.5 equiv) under standard conditions to form the dimer mixture. This was in turn reacted with acetylacetone, under basic conditions, to give the statistical mixture of Ir(ppy)_2_(acac), Ir(ppy≡TIPS)_2_(acac) (**1**), and Ir(ppy≡TIPS)(ppy)(acac) (**2**). This mixture was readily purified by comparatively simple chromatography to give isolated yields of 40% (**1**) and 22% (**2**), comparable to similar synthetic approaches using a mixture of ligands to form the iridium chloride dimer.^11^ Hppy was used as a proof of concept, but these results demonstrate that this approach could be implemented with substituted variations for Hppy. Complexes **1** and **2** were deprotected in situ and coupled via a room-temperature Sonogashira reaction with iodobenzene, 4-iodobenzonitrile, and 4-iodothioanisole to give complexes **3–7** (see Figure 1). The isolated yields for complexes **3–7** ranged from 41% (**7**) to 65% (**4**). These examples demonstrate how this approach can be used to synthesize a divergent range of *tris*-heteroleptic complexes and their complementary *bis*-heteroleptic analogues under mild conditions.

### Structural Analysis

**Figure 2:**
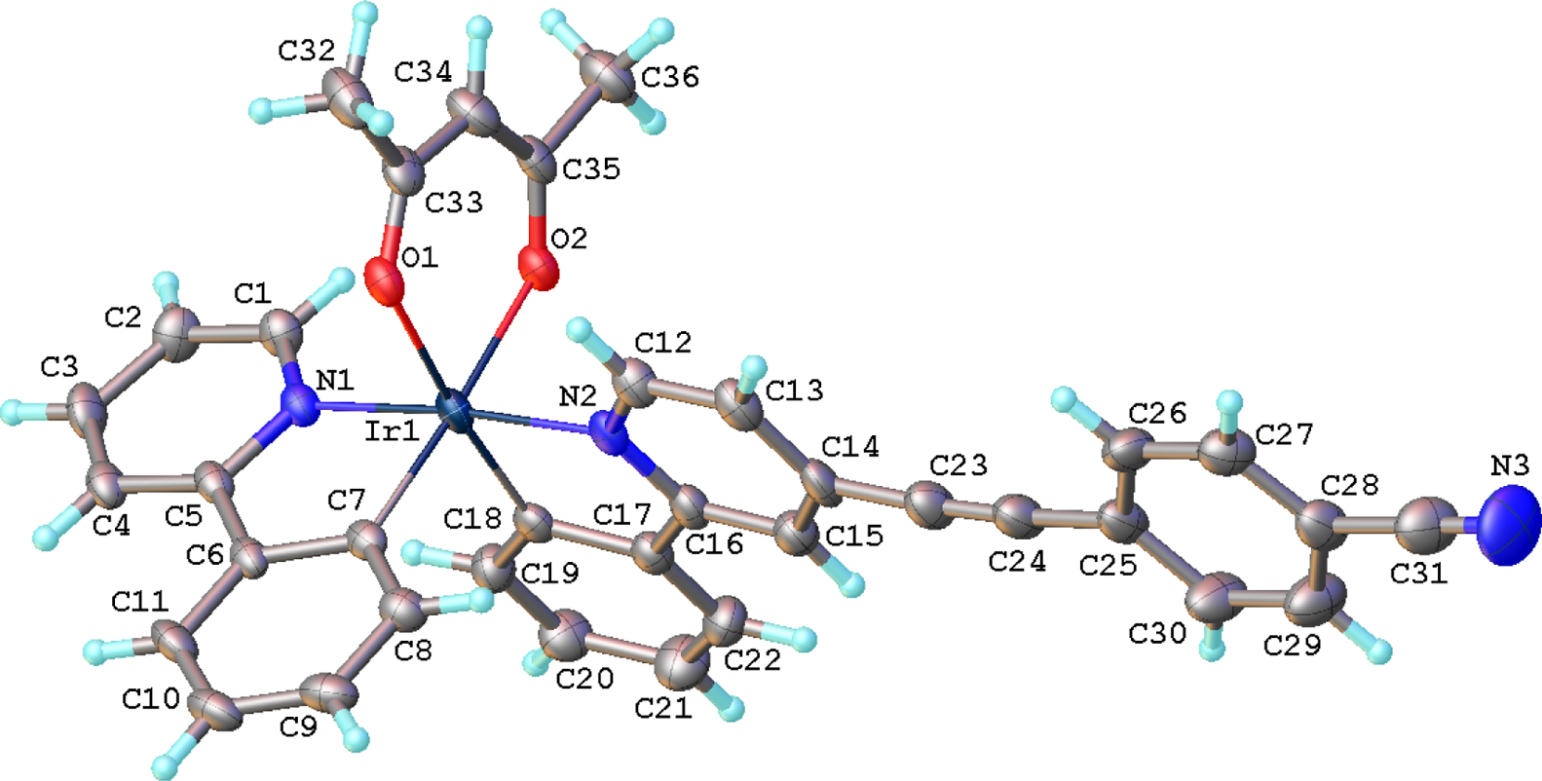
Molecular structure of **6**. Thermal ellipsoids displayed at 50% probability.

The molecular structure determined by X-ray diffraction on a crystal of the *tris*-heteroleptic complex **6** is shown in Figure 2 (CCDC 2169136). Three virtually planar bidentate ligands comprise a typical octahedral coordination of the central iridium atom. The terminal 4-cyanophenylene group is also almost co-planar to the pyridine ring, connected to it via an alkyne; the corresponding dihedral angle is 6.2(1)°. The complexes within the crystal structure are linked together by a variety of weak intermolecular interactions such as CH···N, CH···π, and π···π. The complexes form elliptical channels along the *a*-axis filled with disordered solvent molecules. The narrowest cross section of the channel is approximately 4 Å.

### Electrochemistry

Cyclic voltammograms for complexes **2**, **3**, and **5–7** were recorded in 0.1 M TBAPF_6_ in acetonitrile (MeCN), while the cyclic voltammogram for complex **4** was recorded in 0.1 M TBAPF_6_ in dichloromethane (DCM) owing to its lower solubility (Figures S14–S19 and Table S4). Each of the complexes was internally referenced against ferrocene [i.e., *E*_1/2_(Fc/Fc^+^) = 0.00 V] and displayed a single oxidation event attributed to the characteristic Ir(III)/Ir(IV) couple. The nitrile complexes (**3** and **6**) showed a modest increase in oxidation potential (0.04 and 0.03 V, respectively) relative to the other complexes in either the *bis*- or the *tris*-heteroleptic series. These increases suggest that the nitrile groups are sufficiently electron-withdrawing to impact the HOMO energy, which is typically located at the iridium and cyclometalated phenylene moiety. The potential differences in the nitrile complexes **3** and **6** with respect to other complexes are unexpected as the distance between the nitrile group and the iridium atom is over 11 Å in the structure of **6** (Figure 2).

In addition to the oxidation event, complexes **2**, **3**, and **5**–**7** displayed a reduction event attributed to the reduction of the ligand. For the tris complexes (**5**–**7**), this is most likely to be on the modified ppy ligand given the similar values found with the corresponding *bis*-heteroleptic analogues. Complexes **3** and **6** showed a 0.2 V cathodic shift relative to the other complexes due to the electron-withdrawing nature of the nitrile groups.

### Photophysics

The electronic absorption spectra of the complexes were recorded in DCM (Figures S20, S21). Based on literature comparisons, each of the complexes displayed a ^3^MLCT [metal to ligand charge transfer (MLCT)] band at λ = 450–600 nm, a ^1^MLCT band at λ = 400–350 nm, and a π → π* transition at λ < 350 nm. The MLCT region is broad with a low extinction coefficient (ε), making observable differences between complexes difficult. However, in the π → π* region, the *bis*-heteroleptic complexes (**1**, **3**, and **4**) have ca. double the ε of the *tris*-heteroleptic complexes (**2** and **5**–**7**), which can be attributed to the increased degeneracy of the *bis*-heteroleptic complexes owing to the increased symmetry.

**Figure 3:**
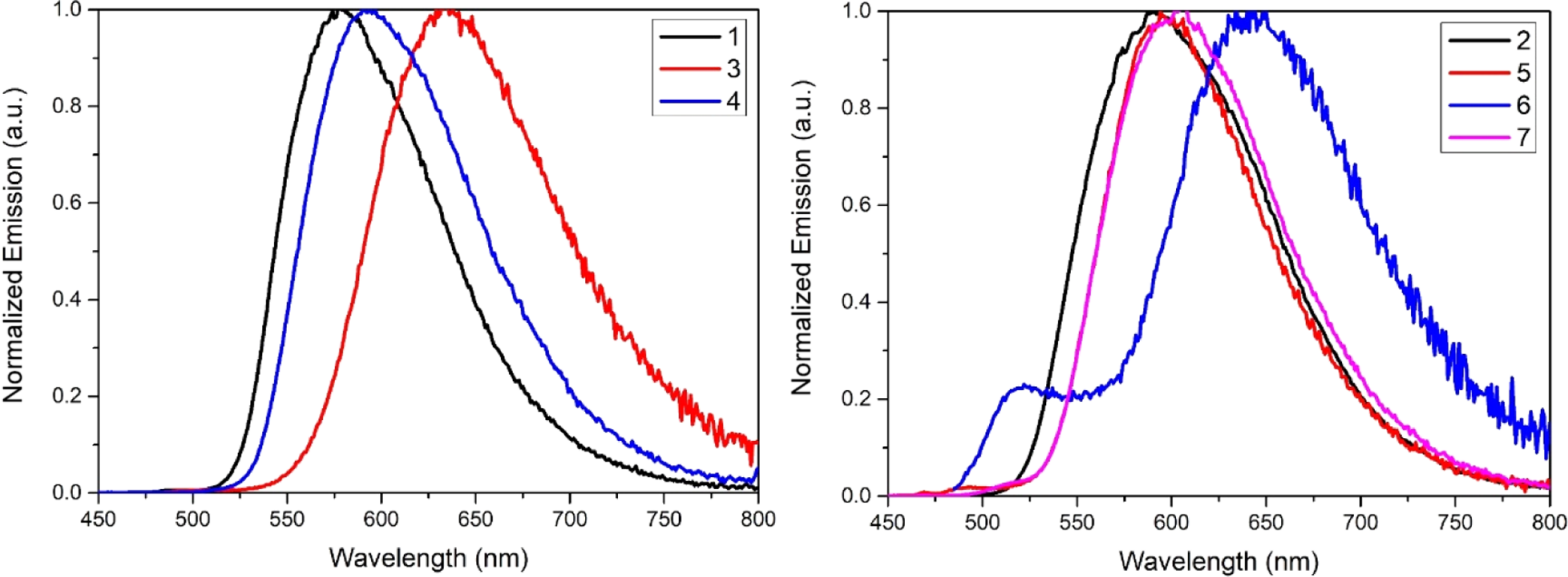
Steady-state emission spectra of *bis*-heteroleptic (left) and *tris-*heteroleptic (right) complexes **1–7** recorded in DCM.

The steady-state emission spectra were recorded in degassed solutions of DCM (see Figure 3, data summarized in Table 1). Each of the complexes (**1**, **2**, **4**, **5**, and **7**) displayed a broad emission at λ = 500–750 nm. Complexes **3** and **6** showed a broad emission at λ = 550–800 nm, significantly red-shifted compared to the previous complexes, due to the strong electron-withdrawing nature of the nitrile groups and the LUMO being localized to the 4-(phenylethynyl)pyridine component of the complex.

**Table 1:** Photophysical Data for Complexes **1–7** Recorded in DCM^a^

complex	λ_emis_	PLQY (Φ)	lifetime (τ, μs)	*k*_r_ × 10^5^ s^–1^	*k*_nr_ ×10^5^ s^–1^	pure radiative lifetime (τ_0_, μs)
Ir(ppy)_2_(acac)^1^	520	0.71	1.90	2.55	3.73	1.52
Ir(ppy≡C_6_H_5_)_2_(acac)^24^^,^^b^	570	0.28	0.93	3.01	7.74	3.32
**1**	580	0.65	1.20	5.42	2.92	1.84
**2**	588	0.69	1.40	4.93	2.21	2.03
**3**	639	0.15	0.22	6.64	38.8	1.51
**4**	600	1.00	0.91	11.0	0	0.91
**5**	603	0.52	0.95	5.47	5.05	1.83
**6**	646	0.086	0.16	5.38	57.1	1.86
**7**	608	0.585	1.10	5.32	3.77	1.88

a The radiative *k*_r_ and non-radiative *k*_nr_ values were calculated according to the equations: *k*_r_ = Φ/τ and *k*_nr_ = (1 – Φ)/τ, from the quantum yields Φ and the lifetime τ values.

b Recorded in toluene.

The *tris*-heteroleptic complexes revealed subtle red shifts in emission relative to their *bis* analogues in solutions; for example, there is a shift from λ_em_ = 600 nm to λ_em_ = 608 nm (Δ*υ* = 219 cm^–1^) when comparing the emission of complexes **4** and **7**. The red shift is attributed to the increased dipole moment associated with the asymmetric structure of the *tris*-heteroleptic complex through lifting of the degeneracy of the two cyclometalated ligands and would be consistent with the ^3^CT [charge transfer (CT)] character of the emission with the relatively short pure-radiative lifetimes of τ_0_ = 0.91 (**4**)–2.03 μs (**2**). As a final means of testing the emission character, the emissions of the complexes were also recorded in cyclohexane, toluene, and acetonitrile solutions. All the complexes displayed a significant red shift in emission as the solvent polarity increased, confirming the emission to be ^3^CT in nature and, given the nature of the complexes, it is specifically a ^3^MLCT emission.

When complexes **3** and **6** were irradiated in DCM over a 60 min period, an additional emissive species was formed with an emission maximum (λ_max_) of 500 nm (Figures S22 and S23). Although the specific nature of this emissive species could not be determined, it is attributed to a product of photodegradation of the initial complexes. This behavior was not observed for other complexes; as such, it must be formed either directly because of the nitrile group or because of its resulting electron deficiency. This photodegradation species appears to form more rapidly for complex **6** than for complex **3**.

A wide range of PLQYs were observed for complexes **1–7**, with the nitrile complexes **3** (Φ = 0.15) and **6** (Φ = 0.086) being low as a result of their high *k*_nr_. In contrast, complex **4** exhibited a high PLQY, Φ = 1.00. Jiang has suggested that the addition of thiomethyl groups to aza-BODIPYs enhances internal CT emissions,^23^ but this warrants further investigation.

### Computational Study

DFT calculations were performed on the optimized ground-state structures for complexes **1–7** with the model chemistry B3LYP/LANL2DZ/3-21G*. This model chemistry has been shown elsewhere to give a reasonable description of the electronic structure of organometallic iridium complexes.^25,26^ A comparison between the fully optimized geometry and the X-ray data for **6** revealed the Ir bond lengths to differ by less than 0.03 Å (Table S5), which gives confidence in the suitability of this model chemistry for complexes **1–7**. The rotations of the aryl end groups attached to the ethynyl unit in **3–7** would be expected to be free in solution at ambient temperature; thus, different conformers of **3–7** present in solutions can influence the photophysical properties, including the absorption spectrum. The rotational barriers were estimated by constraining the aryl end groups to be perpendicular to the pyridyl ring attached to the ethynyl unit and found to be only ca. 4.2 kJ mol^–1^ in all cases. These constrained conformers are denoted with 90° here as in **3**(90, 90°) for **3** and **6**(90°) for **6**.

**Figure 4:**
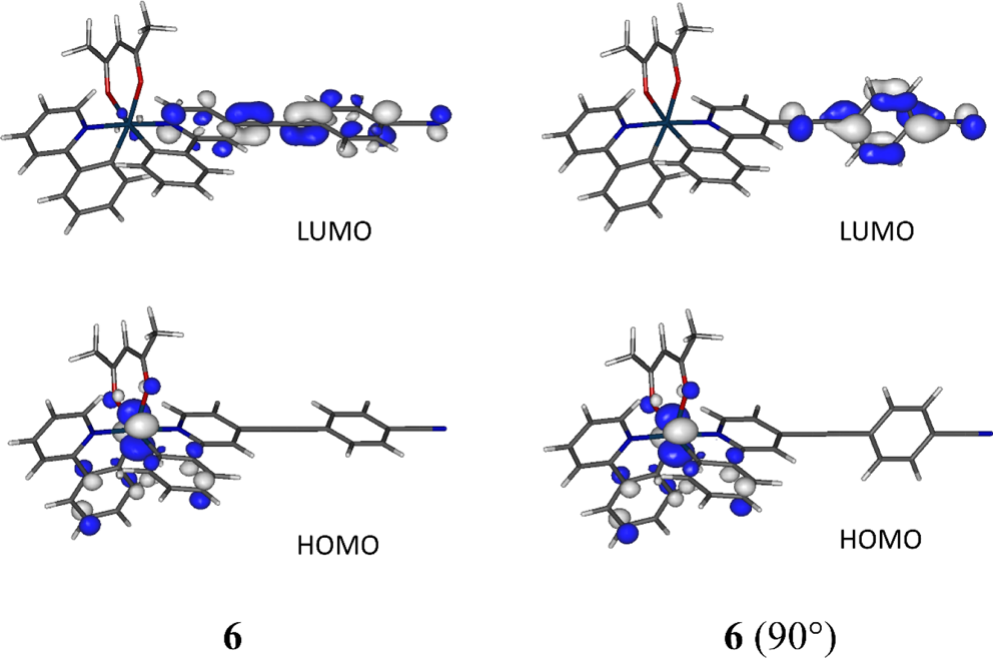
Frontier molecular orbitals for the fully optimized geometry **6** and the constrained geometry **6** (90°). Isocontours at 0.055 e bohr^–3/2^.

Each of the complexes **1–7** showed a HOMO localized to the iridium and both phenylenes of the ppy ligand, as is typical for Ir(ppy)_2_(acac)-based complexes. The HOMO character is independent of the different substitutions made at the 4-position of the pyridyl ring as reflected in the similar observed oxidation potentials of 0.36 to 0.40 V for **1–7**. The HOMO characters remain unchanged with the constrained geometries (Figures 4 and S43–S48). The LUMOs of each of the complexes on the other hand are located on the pyridyl–ethynyl moiety and also on the aryl end groups for **3–7**. For the *bis*-heteroleptic complexes, the LUMO is in a degenerate state with equal contributions from both ligands, likely contributing to the higher PLQYs compared to their *tris*-heteroleptic analogues. The different LUMOs computed are in agreement with the varied reduction potentials observed where the less symmetrical *tris-*heteroleptic complexes **2**, **5**, **6**, and **7** have potentials at −2.33, −2.23, −1.97, and −2.23 V, respectively. This potential trend is in agreement with the trend in their calculated LUMO energies (Table S7). The LUMOs differ between the fully optimized geometries **3–7** and the constrained analogues where the LUMOs are located at the pyridyl**–**ethynyl unit for **4**(90, 90°), **5**(90°), and **7**(90°) and at the aryl end group for **3**(90, 90°) and **6**(90°).

**Figure 5:**
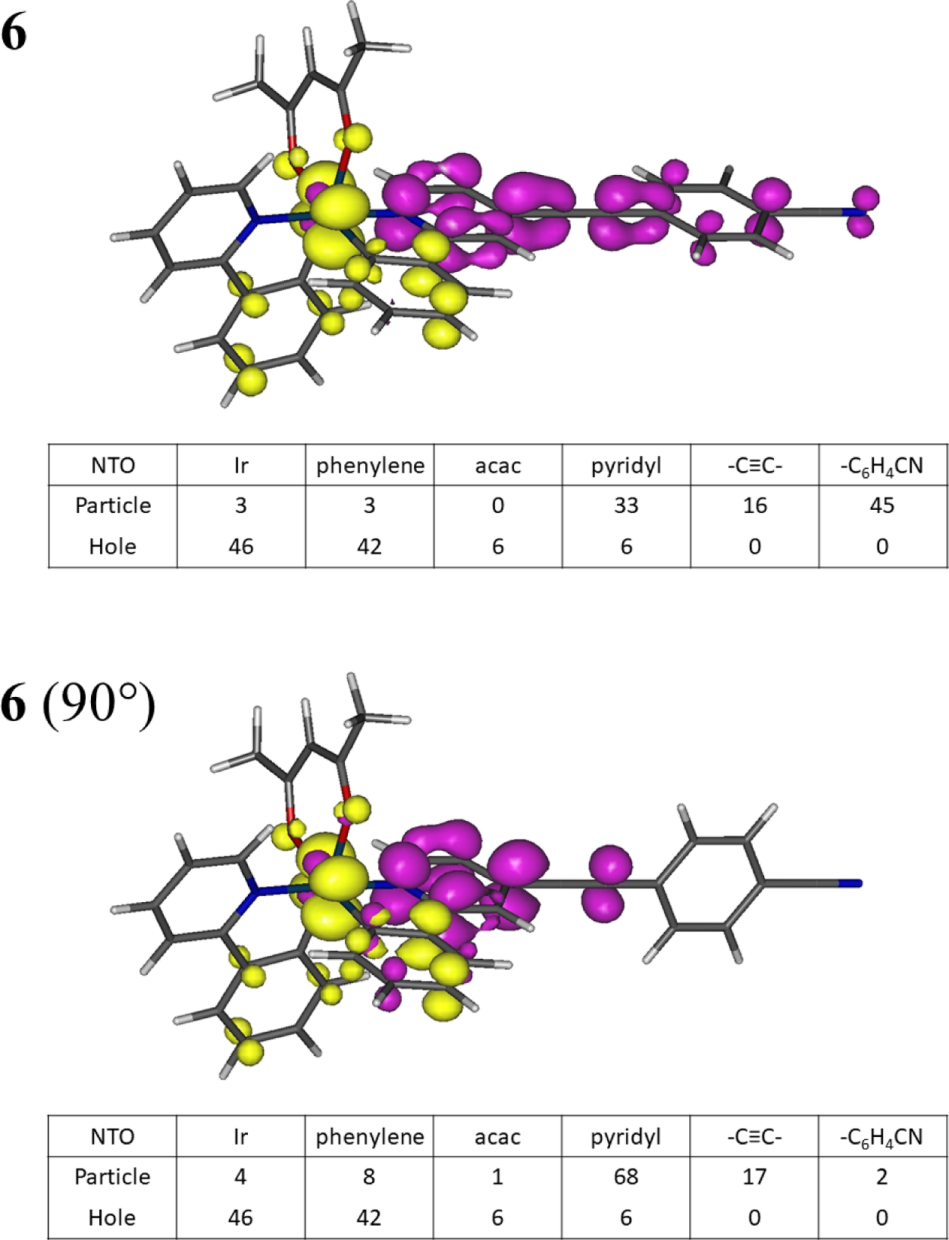
NTOs involved in the S_0_ ← T_1_ emissions for the fully optimized geometry **6** and the constrained geometry **6** (90°). Isocontours at 0.055 e bohr^–3/2^.

Time-dependent DFT calculations confirmed the expected ^3^MLCT character of the S_0_ ← T_1_ transitions for the emissions of all complexes **1–7** based on mirroring the corresponding predicted S_0_ → T_1_ transitions. By adjusting the calculated S_0_ → T_1_ values with a simple scaling factor,^26^ and taking into account both conformers in the case of **3–7**, the agreement with observed emission maxima for all complexes is excellent (Table S8). Natural transition orbitals involved in the NTOs involved in the S_0_ ← T_1_ transitions in all complexes **1–7** show the expected hole orbital at the iridium–phenylene moiety where the phenylene is part of the ethynyl ppy ligand (Figures 5 and S49–S55). The particle orbital is located on the pyridyl–ethynyl moiety in these complexes with some contributions from the aryl end groups for **3–7**, accounting for the emission shift associated with the variation in substitution.

## Conclusions

A versatile and divergent route to synthesizing *tris*-heteroleptic complexes has been demonstrated employing the TIPS-protected synthon complex **2**. This was shown to be readily modified via Sonogashira cross-coupling reactions to produce the *tris*-heteroleptic complexes **5**–**7** and, by utilizing the same approach, their *bis*-heteroleptic analogues could be synthesized from the previously reported TIPS-protected synthon complex **1**. The photophysical properties of both the *tris*- and *bis*-heteroleptic complexes were studied, revealing that each of the complexes had emissions that were ^3^CT in character and significantly red-shifted relative to that of the parent complex Ir(ppy)_2_(acac). Additionally, varying the substituents of these complexes was shown to drastically impact the emission color, lifetime, and PLQY.

## Supporting Information Available

NMR spectra, crystallographic data, photophysical data, and computational details (PDF)
