# Supplementary material for: Divergent Approach for Tris-Heteroleptic Cyclometalated Iridium Complexes Using Triisopropylsilylethynyl-Substituted Synthons
Source: Organometallics. 2022 Aug 19;41(17):2487–93. doi: 10.1021/acs.organomet.2c00292 (PMC9477230; doi:10.1021/acs.organomet.2c00292)
Supplement: Supplementary file 1 — om2c00292_si_001.pdf [file om2c00292_si_001.pdf]

Divergent approach for tris-heteroleptic cyclometalated iridium complexes using triisopropylsilylethynyl substituted synthons

Robert M. Edkins,[b] Yu-Ting Hsu,[a] Mark A. Fox,[a] Dmitry Yufit,[a] Andrew Beeby,[a]* Ross J. Davidson[a]*

[a]*Department of Chemistry, University of Durham, South Road, Durham, DH1 3LE, England, UK*

[b]*WestCHEM Department of Pure and Applied Chemistry, University of Strathclyde, 295 Cathedral Street, Glasgow G1 1XL, UK*

Corresponding Authors

*E-mail: Ross J. Davidson: ross.davidson@durham.ac.uk and Andrew Beeby andrew.beeby@durham.ac.uk

Table of Contents

S1. Synthesis ………………………... S3

S2. NMR spectra of reported compounds ……………………………. S9

S3. Crystallographic data ………………………………………….…..... S16

S3. Electrochemistry …………………………………………........... S20

S5. Photophysical measurements ………………………….............. S24

S6. Calculations …………………………………….......................... S41

S7. References …………………………………......................... S54

S1. Synthesis

General Details

Nuclear magnetic resonance (NMR) spectra were recorded in deuterated solvent solutions on a VNMRS-600 and VNMRS-700spectrometer (Varian Technologies, Palo Alto, CA, USA), and were referenced against solvent resonances (^{1}H, $^{13}C\{^{1}H\}$). Electrospray mass spectra (ESMS) data were recorded on a TQD mass spectrometer (Waters Ltd, UK) in acetonitrile, MALDI–TOF MS data were recorded on an Autoflex II TOF/TOF (Bruker Daltonics, Inc, Billerica, MA, USA). ASAP data were recorded on a Xevo Q-TOF (Waters) high resolution, an accurate mass tandem mass spectrometer equipped with Atmospheric Pressure Gas Chromatography (APGC) and Atmospheric Solids Analysis Probe (ASAP). Microanalyses were performed by Elemental Analysis Service, London Metropolitan University or Durham University Elemental Analysis Service, UK. 2-Phenyl-4-((triisopropylsilyl)ethynyl)pyridine (Hppy-≡-TIPS)[1] and [Ir(ppy-≡-TIPS)$_2$(acac)][1] (**1**) were synthesized according to literature methods. All other chemicals were sourced from standard suppliers.

[Ir(ppy-≡-TIPS)(ppy)(acac)] (2)

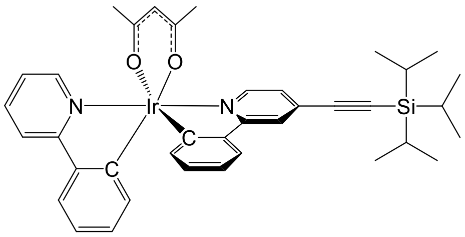

Three compounds, Hppy-≡-TIPS (678 mg, 0.79 mmol), 2-phenylpyridine (0.28 mL, 305 mg, 2.0 mmol), and $IrCl_3·3H_2O$ (715 mg, 2.0 mmol), were suspended in a 3:1 (*v*/*v*) mixture of 2-ethoxyethanol and water (8 mL), and heated at 110 °C for 16 h. During this time, the solution turned from a pale yellow to a dark orange colour. The solution was allowed to cool to room temperature, and water (30 mL) was added, precipitating a bright orange solid, which was filtered, washed with water (2 × 10 mL), and dissolved in CH_2Cl_2 (4 × 20 mL). The CH_2Cl_2 solution was washed with water (10 mL), separated, dried over $MgSO_4$, filtered and reduced *in vacuo* to an orange, oily solid. The crude material was triturated with hexanes (5 mL), yielding a mixture of μ-dichloro-bridged diiridium dimers that appeared as an orange solid. This crude

mixture was dissolved in a 1:1 (*v*/*v*) mixture of acetone:EtOH (30 mL) with acetylacetone (0.5 ml, excess) and K_2CO_3 (250 mg) was suspended in the mixture. The mixture was heated at 60 °C for 4 h. Solvents were removed *in vacuo,* and the residual orange solid was purified by column chromatography (SiO_2, 2:1 (*v*/*v*) CH_2Cl_2:hexanes to CH_2Cl_2), yielding the compound **1** as the fore-running fraction (152 mg, 40%), which appeared as a bright orange solid. The second fraction yielded the title product, also appearing as a bright orange solid. **Yield**: 136 mg (22%). **^{1}H NMR** (700 MHz; $CDCl_3$): δ_H/ppm 8.49 (d, $^3J_{HA,HB}$ = 5.4 Hz, 1H, H_A), 8.46 (d, $^3J_{HA',HB'}$ = 5.9 Hz, 1H, $H_{A'}$), 7.85 (2 H, m), 7.72 (t, $^3J_{HC'HB'HD'}$ = 7.6 Hz, 1H, $H_{C'}$), 7.55 (m, 2H, $H_{E+E'}$), 7.11-7.16 (m, 2H, $H_{B+D'}$), 6.81 (m, 2H, $H_{F+F'}$), 6.70 (m, 2H, $H_{G+G'}$), 6.30 (d, $^3J_{HH,HG}$ = 7.6 Hz, 1H, H_H), 6.26 (d, $^3J_{HH',HG'}$ = 7.6 Hz, 1H, $H_{H'}$), 5.20 (s, 1H, H_I), 1.78 (s, 6H, H_J), 1.19 (m, 21H, H_{K+L}). For information on atom labels, see Figure S1. **^{13}C{^{1}H} NMR** (176 MHz; $CDCl_3$): δ_C/ppm 184.8, 168.7, 168.6, 148.2, 148.1, 147.8, 147.6, 144.8, 144.2, 137.0, 133.2, 132.1, 129.5, 129.3, 124.1, 124.0, 121.6, 121.0, 120.9, 120.8, 118.5, 104.1, 100.5, 98.8, 29.6, 28.9, 18.8, 17.9, 12.4, 11.4. **Acc. MS** ($ASAP^+$): *m/z* 778.2726 (M^+), calcd. for $C_{38}H_{43}N_2O_2Si^{191}Ir$ 778.2700 (|Δ*m*/*z*| = 3.3 ppm). **Elemental Anal.** Calcd. for $C_{38}H_{44}IrN_2O_2Si$: C, 58.43; 5.68; N, 3.59%. Found: C, 58.79; 5.67; N, 3.53%.

General method for Sonogashira coupling with 1

Tetrahydrofuran (50 mL) was added to a suspension containing Et_3N (5 mL), **1** (0.10 mmol), ArI (0.25 mmol), $Pd(PPh_3)_4$ (23 mg, 0.04 mmol), and CuI (4 mg, 0.04 mmol). The solution was degassed via three freeze–pump–thaw cycles, before tetrabutylammonium fluoride (1.0 M in THF, 0.50 mL, 0.50 mmol) was added. The resultant solution was then stirred at room temperature for 12 h before the solvent was removed. The residue was purified by silica chromatography, eluted by dichloromethane (DCM) or a solvent gradient of neat DCM to 1:10 acetone:DCM, and the emissive band was collected. The solvent was removed to yield an orange-red solid.

Ir(PPy-≡-C_6H_4-CN)$_2$(acac) (3)

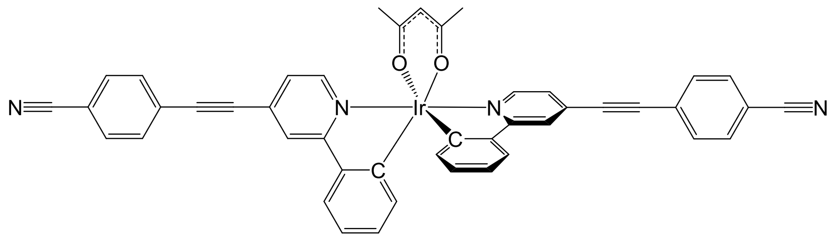

Yield: 41 mg (49%). **^{1}H NMR** (700 MHz, $CDCl_3$): δ_H/ppm 8.51 (d, $^3J_{HA,HB}$ = 5.9 Hz, 2H, H_A), 7.94 (s, 2H, H_C), 7.71-7.68 (m, 8H, H_{H+I}), 7.57 (dd, $^3J_{HG,HF}$ = 7.9 Hz, $^4J_{HG,HE}$ = 1.4 Hz), 2H, H_G), 7.20 (dd, $^3J_{HB,HA}$ = 5.9 Hz, $^4J_{HB,HC}$ = 1.8 Hz, 2H, H_B), 6.83 (td, $^3J_{HF,HE,HG}$ = 7.5 Hz, $^4J_{HF,HD}$ = 1.2 Hz, 2H, H_F), 6.72 (td, $^3J_{HE,HD,HF}$ = 7.4 Hz, $^4J_{HE,HG}$ = 1.3 Hz, 2H, H_E), 6.28 (dd ($^4J_{HD,HE}$ = 7.7 Hz, $^4J_{HD,HF}$ = 1.2 Hz, 2H, H_D), 5.23 (s, 1H, H_J), 1.80 (s, 6H, H_K). For information on atom labels, see Figure S3. **^{13}C{^{1}H} NMR** (176 MHz; $CDCl_3$): δ_C/ppm 184.9, 168.6, 147.8, 147.6, 143.6, 133.0, 132.4, 132.2, 131.0, 129.5, 126.7, 124.1, 123.3, 121.0, 120.4, 118.1, 112.7, 100.5, 93.3, 90.3, 28.7. **Acc. MS** (ASAP$^+$): *m/z* 849.1947 [M]$^+$, calcd. for $C_{45}H_{30}N_4O_2{}^{191}Ir$ 849.1975 (|Δ*m*/*z*| = 3.3 ppm).

Ir(PPy-≡-C_6H_4-SMe)$_2$(acac) (4)

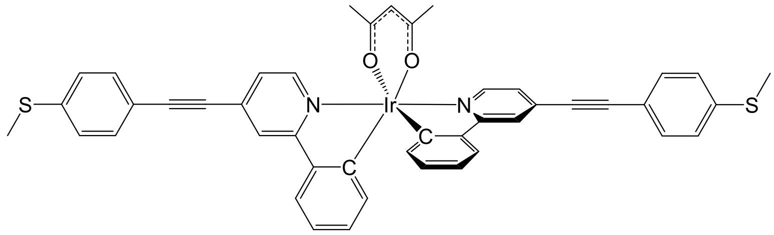

Yield: 60 mg (65%). **^{1}H NMR** (600 MHz, TCE-d_2): δ_H/ppm 8.45 (d, $^3J_{HA,HB}$ = 5.9 Hz, 2H, H_A), 7.95 (s, 2H, H_c), 7.64 (dd, $^3J_{HG,HF}$ = 7.4 Hz, $^4J_{HG,HE}$ = 1.5 Hz, 2H, H_G), 7.57 (d, $^4J_{HI,HH}$ = 8.3 Hz, 4H, H_I), 7.29-7.28 (m, 6H, H_{B+H}), 6.95 (td, $^3J_{HF,HG,HE}$ = 7.4 Hz, $^4J_{HF,HD}$ = 1.5 Hz, 2H, H_F), 6.78 (td, $^3J_{HE,HD,HF}$ = 7.4 Hz, $^4J_{HE,HG}$ = 1.5 Hz, 2H, H_E), 6.32 (dd, $^3J_{HD,HE}$ = 7.7 Hz, $^4J_{HD,HF}$ = 1.2 Hz, 2H, H_D), 5.30 (s, 1H, H_J), 2.55 (s, 6H, H_L), 1.86 (s, 6H, H_K). For information on atom labels, see Figure S5. **^{13}C{^{1}H} NMR** (150 MHz, $CDCl_3$): δ_C/ppm 184.8, 168.3, 147.6, 147.9, 144.0, 141.1, 133.0, 132.2, 132.1, 129.3, 125.7, 123.9, 123.2, 120.9, 120.2, 117.9, 100.4, 95.6, 86.8,

28.7, 15.1.[i] **MS-MALDI**: *m/z* 829.1 [M$^+$]. **Elemental Anal.** Calcd. for $C_{45}H_{35}IrN_2O_2S_2$: C, 60.59; H, 3.95; N, 3.14%. Found: C, 60.53; H, 3.96; N, 3.11%.

General method for Sonogashira coupling with 2

Tetrahydrofuran (50 mL) was added to a suspension containing Et_3N (5 mL), **3** (0.10 mmol), ArI (0.15 mmol), $Pd(PPh_3)_4$ (11 mg, 0.01 mmol), and CuI (1.9 mg, 0.01 mmol). The resultant solution was degassed by three freeze–pump–thaw cycles, before tetrabutylammonium fluoride (1.0 M in THF, 0.25 mL, 0.25 mmol) was added. The solution was then stirred at room temperature for 12 h before the solvent was removed. The residue was purified by silica chromatography eluted by either 1:2 hexane:DCM or neat DCM, collecting the emissive band. The solvent was removed to yield an orange solid.

Ir(ppy)(ppy-≡-C_6H_5)(acac) (5)

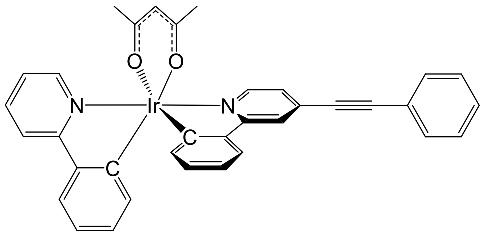

Yield: 38 mg (55%). **^{1}H NMR** (700 MHz; $CDCl_3$): δ_H/ppm 8.50-8.47 (m, 2H, $H_{A+A'}$), 7.93 (d, $^4J_{HD,HB}$ = 1.9 Hz, 1H, H_D), 7.84 (dd, $^3J_{HD',HC'}$ = 8.3 Hz, $^4J_{HD',HB'}$ = 1.1 Hz, 1H, $H_{D'}$), 7.72 (td, $^3J_{HC',HB',HD'}$ = 8.2 Hz, $^4J_{HC',HA'}$ = 1.6 Hz, 1H, $H_{C'}$), 7.62-7.60 (m, 2H, H_K), 7.56 (dd, $^3J_{HE,HF}$ = 7.7 Hz, $^4J_{HE,HG}$ = 1.3 Hz, 1H, H_E), 7.54 (dd, $^3J_{HE',HF'}$ = 7.7 Hz, $^4J_{HE,HG}$ = 1.3 Hz, 1H, $H_{E,}$), 7.43-7.40 (m, 3H, H_{L+M}), 7.18 (dd, $^3J_{HB,HA}$ = 5.9 Hz, $^4J_{HB,HD}$ = 1.8 Hz, 1H, H_B), 7.13 (td, $^3J_{HB',HA',Hc'}$ = 7.3 Hz, $^4J_{HB',HD'}$ = 1.4 Hz, 1H, $H_{B'}$), 6.83-6.78 (m, 2H, $H_{F+F'}$), 6.73-6.68 (m, 2H, $H_{G+G'}$), 6.30 (dd, $^3J_{HH,HG}$ = 7.7 Hz, $^4J_{HH,HF}$ = 1.2 Hz, 1H, H_H), 6.27 (dd, $^3J_{HH',HG'}$ = 7.7, $^4J_{HH',HF'}$ = 1.1 Hz, 1H, $H_{H'}$), 5.21 (s, 1H, H_I), 1.79 (s, 6H, H_J). For information on atom labels, see Figure S8. **^{13}C{^{1}H} NMR** (176 MHz; $CDCl_3$): δ_C/ppm 184.6, 168.5, 168.4, 148.0, 147.9, 147.4, 147.4, 144.6, 144.0, 136.8, 133.0, 132.0, 131.9, 129.3, 129.1, 128.5, 123.9, 123.7,

[i] Limited solubility of the compound gave poor ^{13}C NMR data.

123.2, 120.3, 118.3, 100.3, 95.5, 85.6, 28.7. **MS-MALDI**: *m/z* 700.2 [M$^+$]. **Acc. MS** (ASAP$^+$): *m/z* 699.1778 [M]$^+$, calcd. for $C_{35}H_{28}N_2O_2{}^{191}Ir$ 699.1757 (|Δ*m/z*| = 1.8 ppm)

Ir(PPy)(PPy-≡-C$_6$H$_4$-CN)(acac) (6)

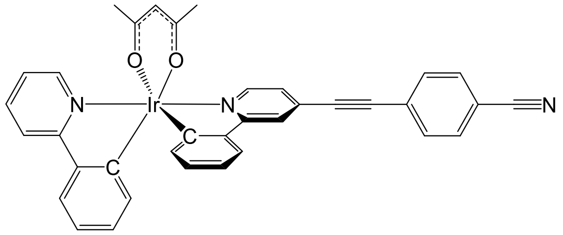

Yield: 42 mg (59%). **^{1}H NMR** (700 MHz; TCE-d$_2$): δ_H/ppm 8.50 (d, $^3J_{HA,HB}$ = 5.9 Hz, 1H, H_A), 8.46 (d, $^3J_{HA',HB'}$ = 5.9 Hz, 1H, $H_{A'}$), 7.95 (d, $^3J_{HD,HB}$ = 1.8 Hz, 1H, H_D), 7.86 (dd, $^3J_{HD',HC'}$ = 8.2 Hz, $^4J_{HD',HB'}$ = 1.2 Hz, 1H, $H_{D'}$), 7.79 (td, $^3J_{HC',HB',HD'}$ = 7.9 Hz, $^4J_{HC',HA'}$ = 1.6 Hz, 1H, $H_{C'}$), 7.75-7.72 (m, 4H, H_{K+L}), 7.63 (dd, $^3J_{HE,HF}$ = 7.7 Hz, $^4J_{HE,HG}$ = 1.3 Hz, 1H, H_E), 7.60 (dd, $^3J_{HE',HF'}$ = 7.7 Hz, $^4J_{HE',HG'}$ = 1.3 Hz, 1H, $H_{E'}$), 7.28 (dd, $^3J_{HB,HA}$ = 5.9 Hz, $^4J_{HB,HD}$ = 1.8 Hz, 1H, H_B), 7.32 (ddd, $^3J_{HB',HC'}$ = 7.2 Hz, $^3J_{HB',HA'}$ = 5.7 Hz, $^4J_{HB',HD'}$ = 1.4 Hz, 1H, $H_{B'}$), 6.95-6.92 (m, 2H, $H_{F+F'}$), 6.77-6.74 (m, 2H, $H_{G+G'}$), 6.27 (dd, $^3J_{HH/',HG/'}$ = 7.7 Hz, $^4J_{HH/',HF/'}$ = 1.2 Hz, 2H, $H_{H+H'}$), 5.28 (s, 1H, H_I), 1.84 (s, 6H, H_J). For information on atom labels, see Figure S10. **^{13}C{^{1}H} NMR** (176 MHz; TCE-d$_2$): δ_C/ppm 185.0, 184.9, 168.4, 167.8, 148.1, 148.0, 147.6, 146.7, 145.3, 144.5, 137.4, 133.7, 133.5, 132.6, 132.3, 131.0, 129.2, 128.8, 126.7, 124.3, 124.0, 123.9, 122.0, 120.9, 120.5, 118.6, 112.5, 101.1, 93.7, 90.4, 29.0. **Acc. MS** (ASAP$^+$): *m/z* 723.1624 [M]$^+$, calcd. for $C_{36}H_{26}N_3O_2{}^{191}Ir$ 723.1631 (|Δ*m*/z| = 1.5 ppm).

Ir(PPy)(PPy-≡-C$_6$H$_4$-SMe)(acac) (7)

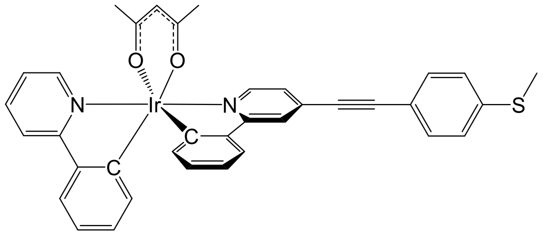

Yield: 30 mg (41%). **^{1}H NMR** (700 MHz; CDCl$_3$): δ_H/ppm 8.49 (d, $^3J_{HA,HB}$ = 5.73 Hz, 1H, H_A), 8.47 (d, $^3J_{HA',HB'}$ = 5.9 Hz, 1H, $H_{A'}$), 7.90 (d, $^4J_{HD,HB}$ = 1.6 Hz, 1H, H_D), 7.84 (d, $^3J_{HD',HC'}$ = 8.0 Hz, 1H, $H_{D'}$), 7.72 (td, $^3J_{HC',HD',HB'}$ = 7.8 Hz, $^4J_{HC',HA'}$ = 1.6 Hz, 1H, $H_{C'}$), 7.56-

7.50 (m, 6H), 7.16 (dd, $^{3}J_{HB,HA}$ = 5.9 Hz, $^{4}J_{HB,HD}$ = 1.8 Hz, 1H, H_{B}), 7.13 (td, $^{3}J_{HB',HA'HC'}$ = 7.2 Hz, $^{4}J_{HB',HD'}$ = 1.4 Hz, 1H, $H_{B'}$), 6.82-6.79 (m, 2H, $H_{F+F'}$), 6.70-6.68 (m, 2H, $H_{G+G'}$), 6.29 (d, $^{3}J_{HH,HG}$ = 7.6 Hz, 1H, H_{H}), 6.26 (d, $^{3}J_{HH',HG'}$ = 7.6 Hz, 1H, $H_{H'}$), 5.20 (s, 1H, H_{I}), 2.52 (s, 3H, H_{M}), 1.78 (pseudo doublet, 6H, H_{J}). For information on atom labels, see Figure S12. **^{13}C{^{1}H} NMR** (176 MHz; $CDCl_3$): δ_{C}/ppm 184.9, 168.4, 167.8, 148.0, 147.6, 146.7, 145.3, 144.5, 137.4, 133.7, 133.5, 132.6, 132.3, 131.0, 129.2, 128.8, 126.7, 124.3, 124.0, 123.9, 122.0, 120.9, 120.5, 118.6, 112.5, 101.1, 93.7, 90.4, 28.7. **Acc-MS** (ASAP^{+}): *m/z* 745.1661 $[M]^{+}$, calcd. for $C_{36}H_{29}N_{2}O_{2}S^{191}Ir$ 745.1634 (|Δ*m*/*z*| = 3.6 ppm). $^{+}$): *m/z* 645.1115 $[M–acac]^{+}$, calcd. for $C_{31}H_{22}N_{2}S^{191}Ir$ 645.1110 (|Δ*m*/*z*| = 0.8 ppm).

S2. NMR spectra of reported compounds

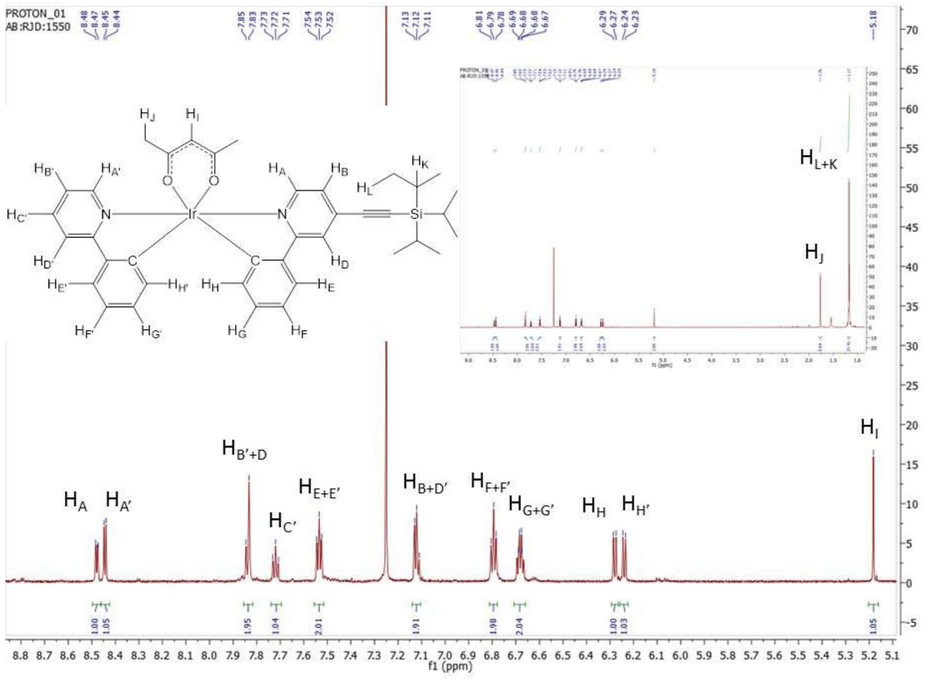

Figure S1. ^{1}H NMR spectrum of **2**, recorded in $CDCl_3$.

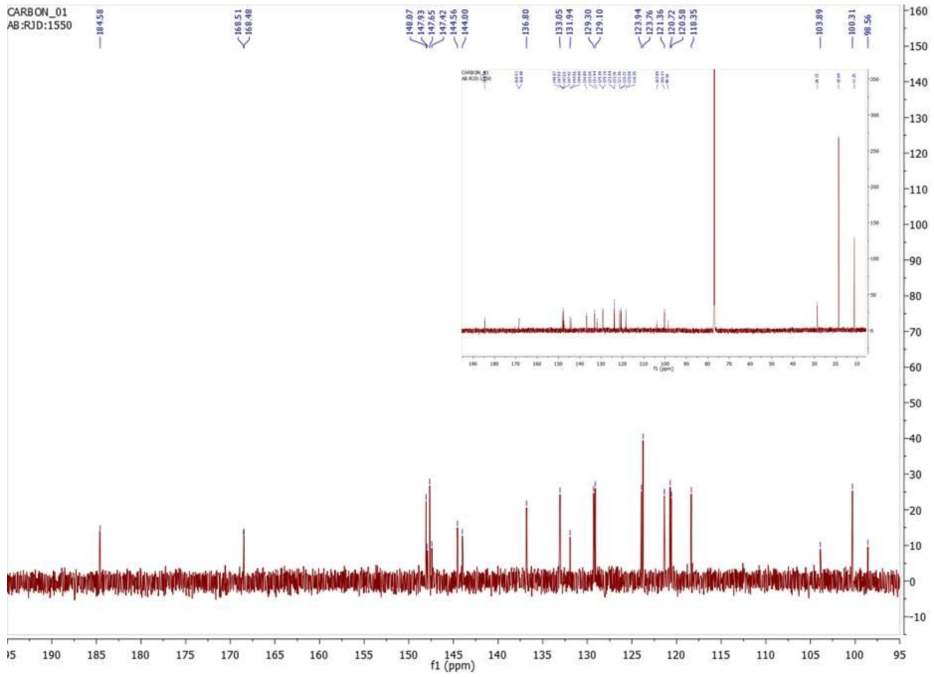

Figure S2. $^{13}C\{^{1}H\}$ NMR spectrum of **2**, recorded in $CDCl_3$.

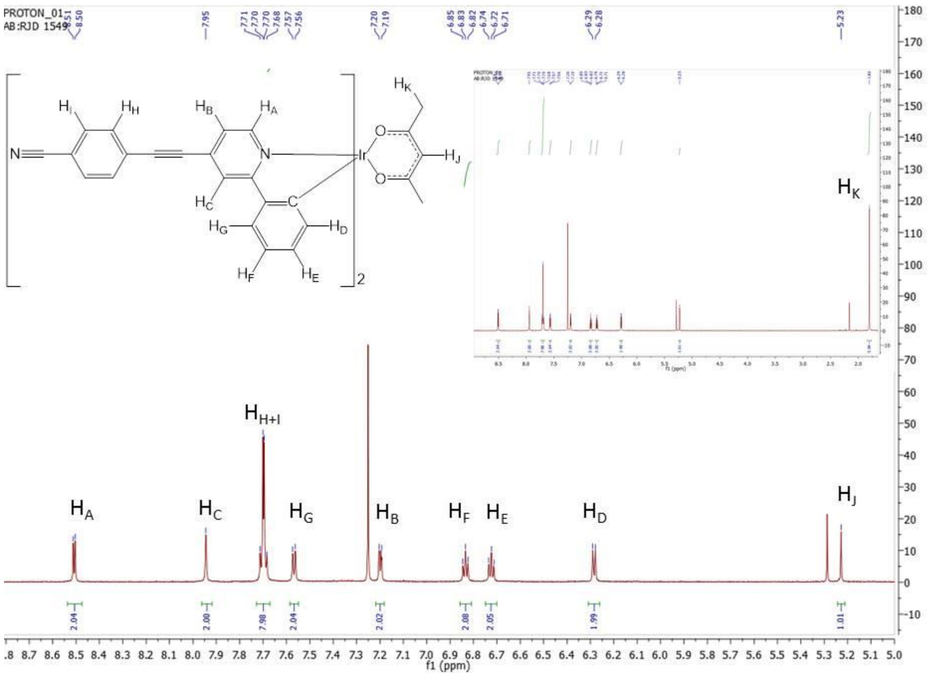

Figure S3. ^{1}H NMR spectrum of **3**, recorded in $CDCl_3$.

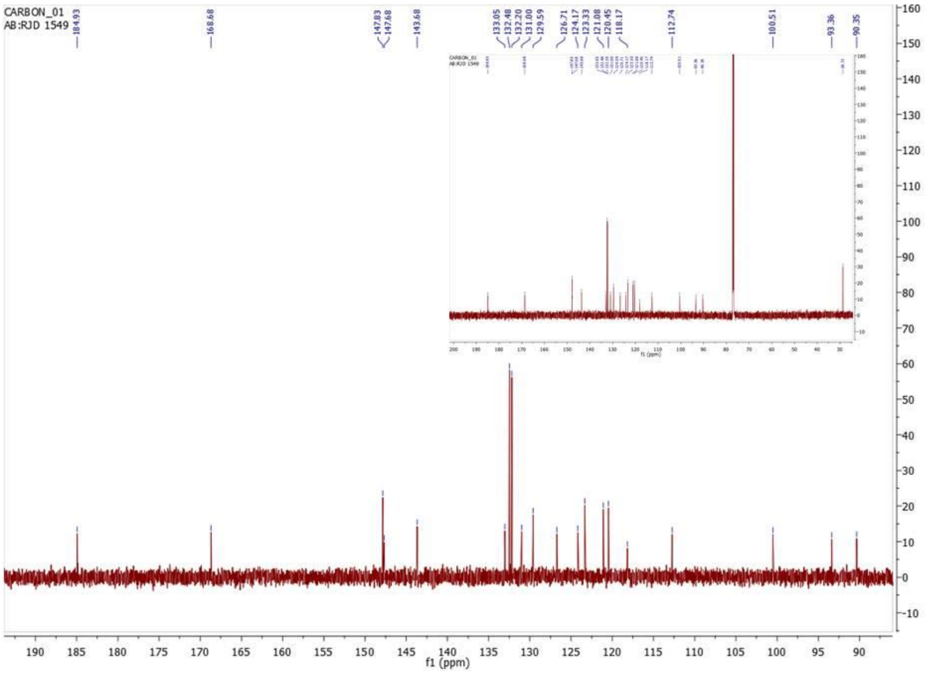

Figure S4. $^{13}C\{^{1}H\}$ NMR spectrum of **3**, recorded in $CDCl_3$.

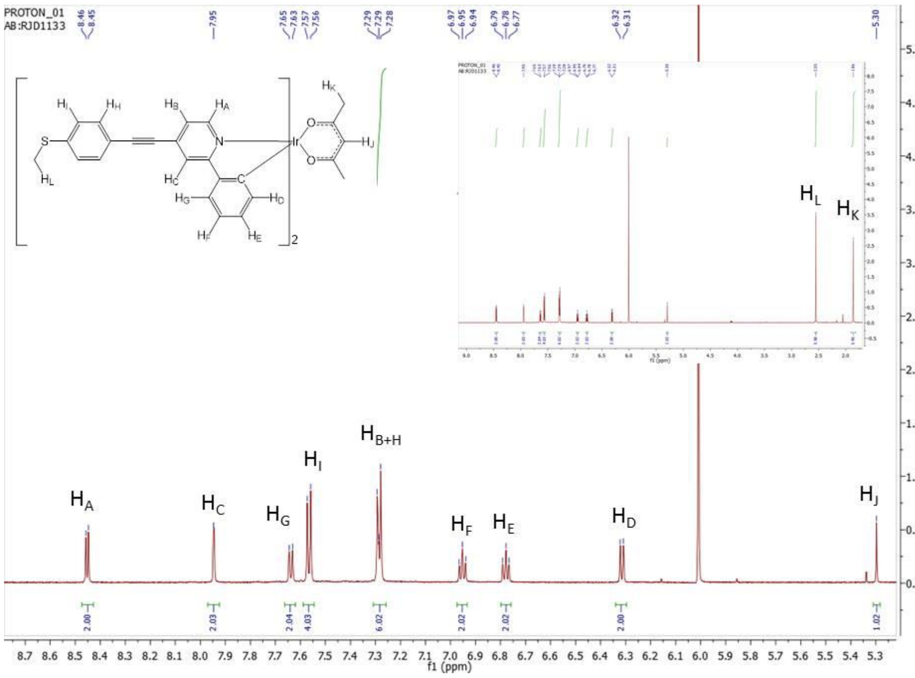

Figure S5. ^{1}H NMR spectrum of **4**, recorded in TCE-d_2.

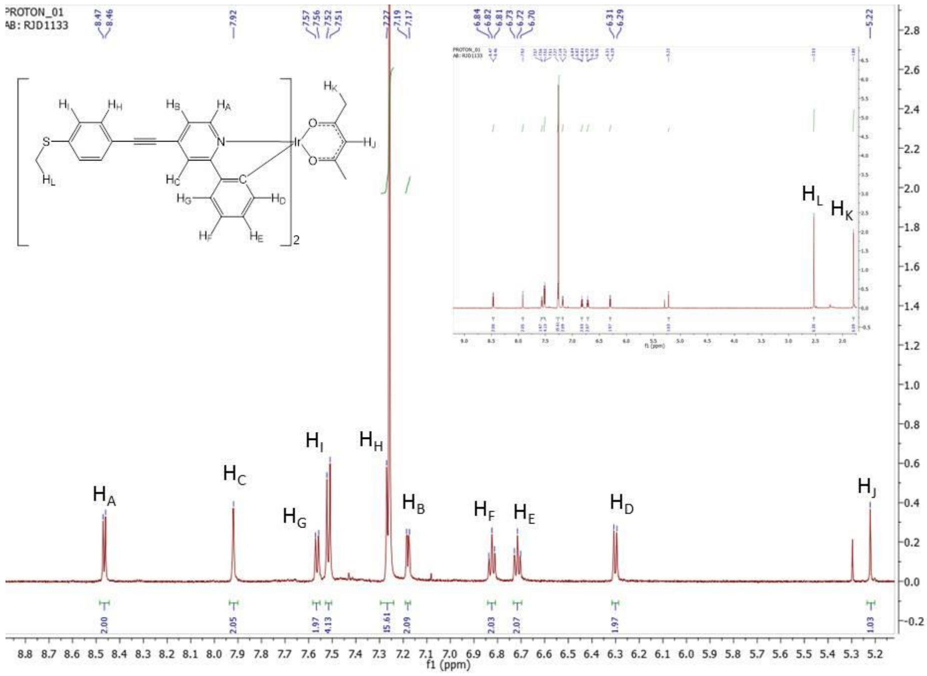

Figure S6. ^{1}H NMR spectrum of **4**, recorded in $CDCl_3$.

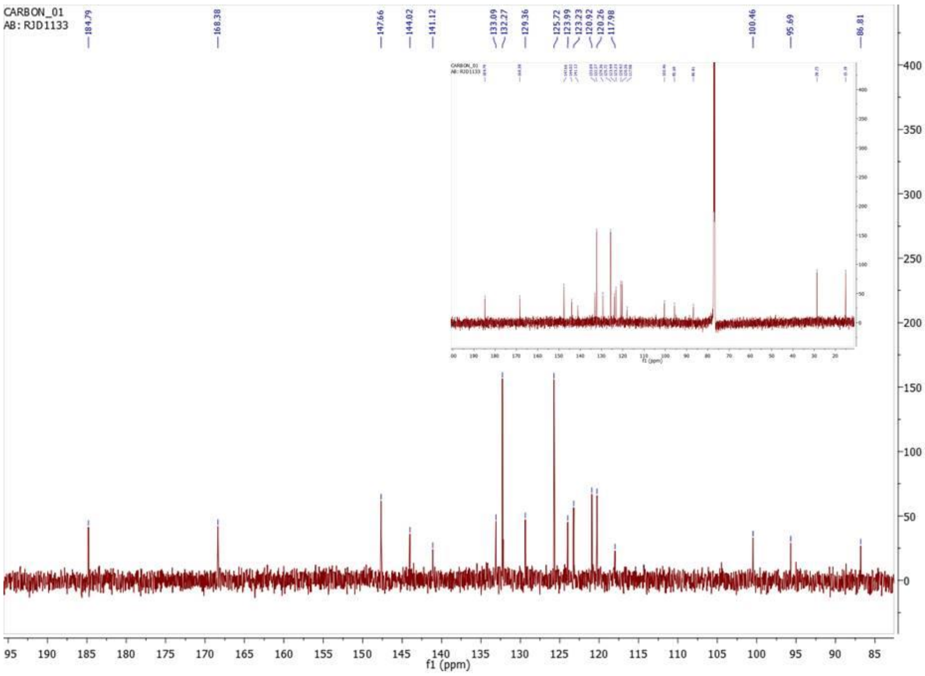

Figure S7. $^{13}C\{^{1}H\}$ NMR spectrum of **4**, recorded in $CDCl_3$.

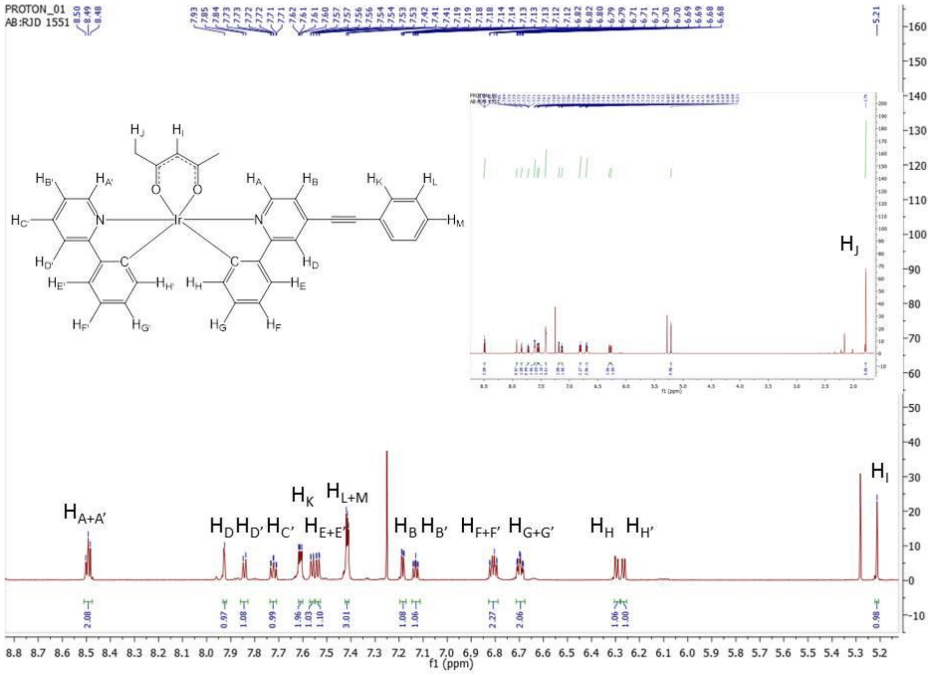

Figure S8. ^{1}H NMR spectrum of **5**, recorded in $CDCl_3$.

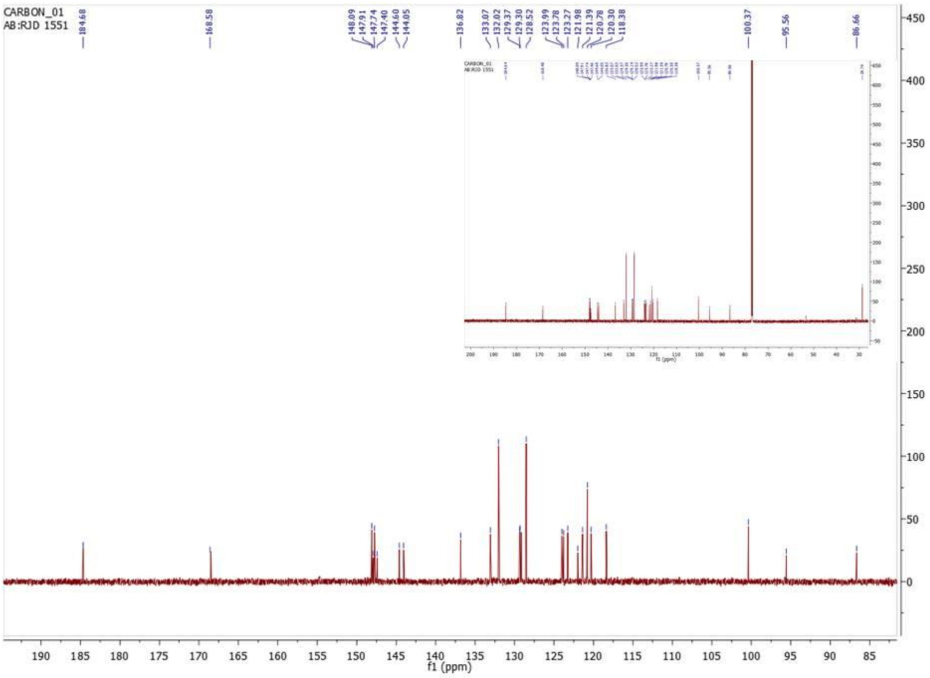

Figure S9. ${}^{13}C\{{}^{1}H\}$ NMR spectrum of **5**, recorded in $CDCl_3$.

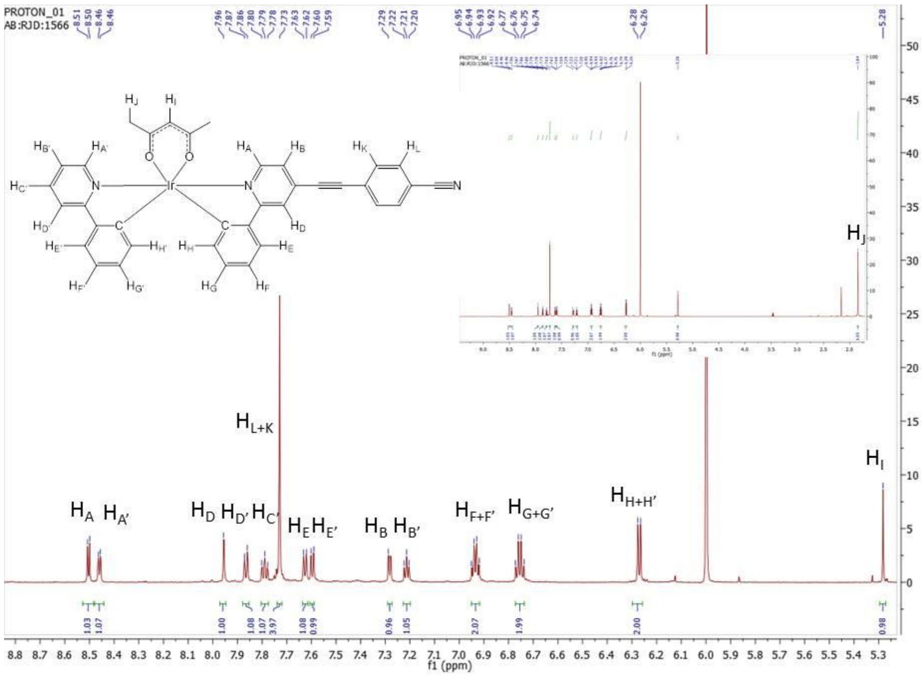

Figure S10. ${}^{1}H$ NMR spectrum of **6**, recorded in TCE-d_2.

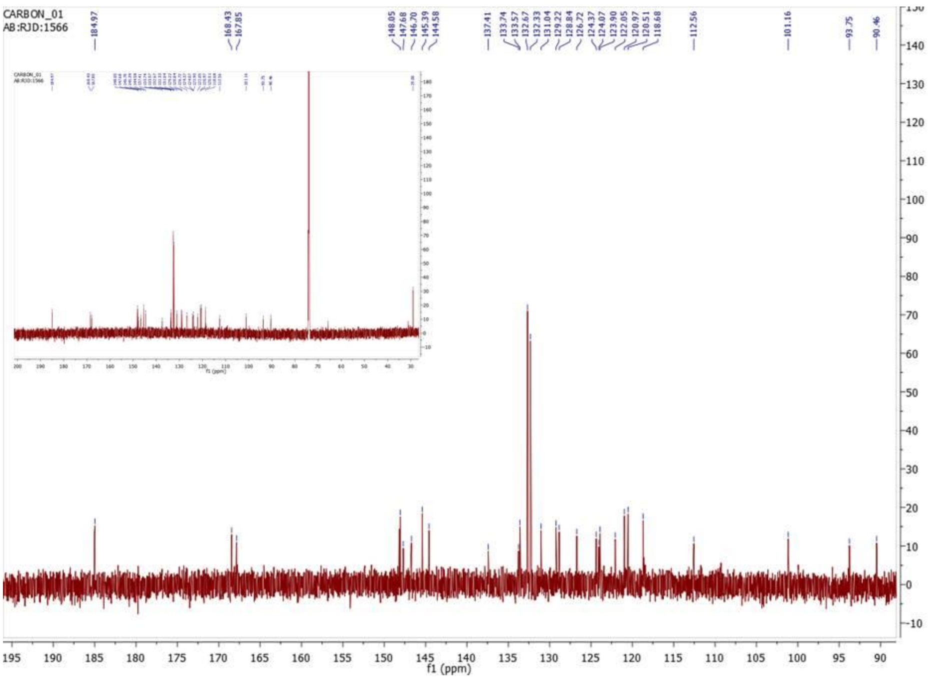

Figure S11. $^{13}C\{^1H\}$ NMR spectrum of **6**, recorded in TCE-d_2.

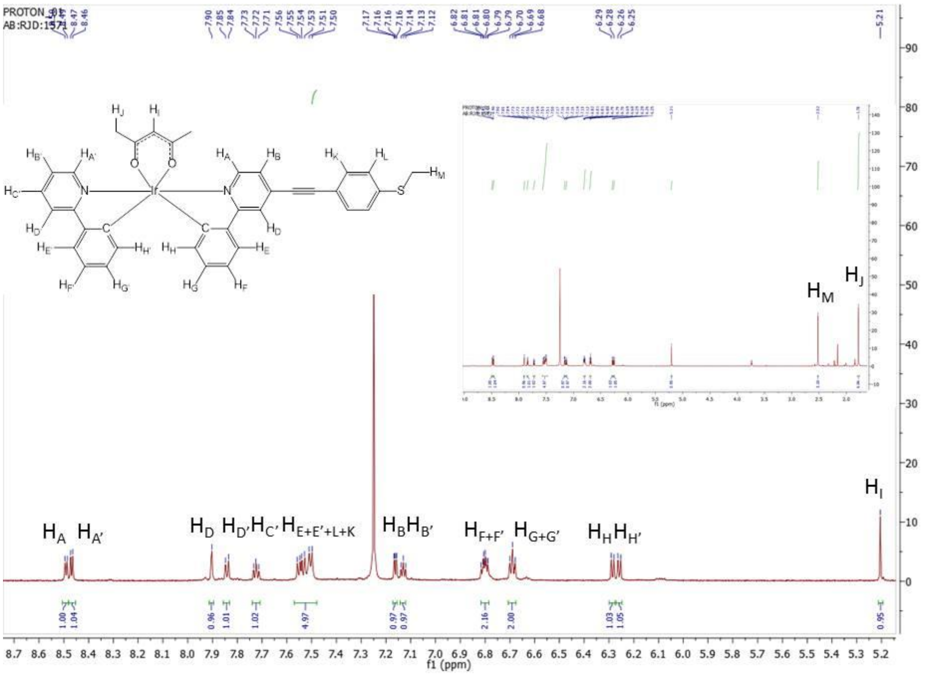

Figure S12. ^1H NMR spectrum of **7**, recorded in $CDCl_3$.

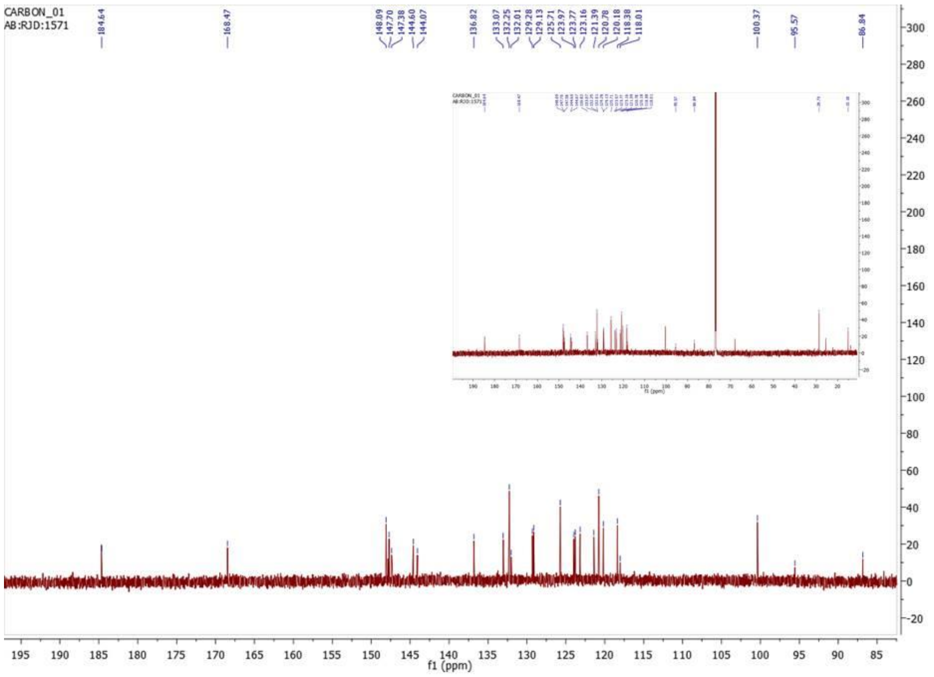

Figure S13. ${}^{13}C\{{}^{1}H\}$ NMR spectrum of **7**, recorded in $CDCl_3$.

S3. Crystallographic data

The X-ray single crystal data have been collected using $\lambda_{MoK\alpha}$ radiation (λ = 0.71073 Å) on a Bruker D8Venture (Photon100 CMOS detector, IμS-microsource, focusing mirrors) diffractometer equipped with a Cryostream (Oxford Cryosystems) open-flow nitrogen cryostats at the temperature 120.0(2) K. The structure was solved by direct method and refined by full-matrix least squares on F^2 for all data using Olex2[2] and SHELXTL[3] software. All non-hydrogen atoms were refined in anisotropic approximation; hydrogen atoms were placed in the calculated positions and refined in riding mode. The structure of **6** contains several severely disordered solvent molecules, most probably three DMF molecules, which could not be modelled and refined reliably. The contribution of disordered solvent molecules into diffraction intensities was taken into account by application of the MASK procedure of the Olex2 program package.

Crystal data and parameters of refinement are listed in Table S1. Crystallographic data for the structure have been deposited with the Cambridge Crystallographic Data Centre as supplementary publication CCDC-2169136.

Table S1. Crystal and Refinement Data for **6**

**Compound**	**6**
Empirical formula	$C_{36}H_{26}IrN_3O_2$
Formula weight	724.80
Temperature/K	120.0
Crystal system	triclinic
Space group	*P*-1
a/Å	9.7255(5)
b/Å	11.4855(6)
c/Å	14.8982(8)
α/°	103.6151(19)
β/°	92.9966(19)
γ/°	94.0713(19)
Volume/Å^3	1609.23(15)
Z	2
ρ_{calc}g/cm^3	1.496
μ/mm^{-1}	4.183
F(000)	712.0
Crystal size/mm^3	0.27 × 0.13 × 0.03
Radiation	MoKα (λ = 0.71073)
2Θ range for data collection/°	4.21 to 57.998
Index ranges	$-13 \leq h \leq 13$, $-15 \leq k \leq 15$, $-20 \leq l \leq 20$
Reflections collected	33240
Independent reflections	8560 [R_{int} = 0.0447, R_{sigma} = 0.0475]
Data/restraints/parameters	8560/0/381
Goodness-of-fit on F^2	1.028
Final R indexes [$I \geq 2\sigma(I)$]	R_1 = 0.0338, wR_2 = 0.0731
Final R indexes [all data]	R_1 = 0.0455, wR_2 = 0.0768
Largest diff. peak/hole / e Å^{-3}	2.35/–0.78

Table S2. Selected bond lengths for **6**.

Atom	Atom	Length/Å		Atom	Atom	Length/Å
Ir1	O1	2.164(2)		C13	C14	1.396(6)
Ir1	O2	2.142(2)		C14	C15	1.393(5)
Ir1	N1	2.034(3)		C14	C23	1.427(5)
Ir1	N2	2.024(3)		C15	C16	1.389(5)
Ir1	C7	1.993(3)		C16	C17	1.456(5)
Ir1	C18	1.981(4)		C17	C18	1.419(5)
O1	C33	1.267(4)		C17	C22	1.393(5)
O2	C35	1.266(4)		C18	C19	1.397(5)
N1	C1	1.351(4)		C19	C20	1.389(5)
N1	C5	1.362(4)		C20	C21	1.397(6)
N2	C12	1.346(4)		C21	C22	1.387(5)
N2	C16	1.372(5)		C23	C24	1.188(5)
N3	C31	1.119(6)		C24	C25	1.442(5)
C1	C2	1.363(5)		C25	C26	1.387(5)
C2	C3	1.392(5)		C25	C30	1.391(6)
C3	C4	1.371(5)		C26	C27	1.367(5)
C4	C5	1.390(5)		C27	C28	1.390(6)
C5	C6	1.456(5)		C28	C29	1.394(6)
C6	C7	1.416(5)		C28	C31	1.444(6)
C6	C11	1.396(5)		C29	C30	1.375(6)
C7	C8	1.403(5)		C32	C33	1.509(5)
C8	C9	1.382(5)		C33	C34	1.402(5)
C9	C10	1.378(6)		C34	C35	1.389(5)
C10	C11	1.390(5)		C35	C36	1.496(5)
C12	C13	1.368(5)				

Table S3. Selected bond angles for **6**.

Atom	Atom	Atom	Angle/°		Atom	Atom	Atom	Angle/°
O2	Ir1	O1	88.42(9)		N2	C12	C13	122.7(4)
N1	Ir1	O1	89.86(10)		C12	C13	C14	119.3(3)
N1	Ir1	O2	95.55(10)		C13	C14	C23	119.2(4)
N2	Ir1	O1	94.38(11)		C15	C14	C13	118.3(3)
N2	Ir1	O2	87.87(10)		C15	C14	C23	122.4(4)
N2	Ir1	N1	174.64(10)		C16	C15	C14	120.3(4)
C7	Ir1	O1	90.54(11)		N2	C16	C15	120.1(3)
C7	Ir1	O2	176.03(11)		N2	C16	C17	113.7(3)
C7	Ir1	N1	80.62(13)		C15	C16	C17	126.1(3)
C7	Ir1	N2	96.03(13)		C18	C17	C16	114.6(3)
C18	Ir1	O1	174.63(11)		C22	C17	C16	123.3(3)
C18	Ir1	O2	88.58(11)		C22	C17	C18	122.0(3)
C18	Ir1	N1	94.86(13)		C17	C18	Ir1	114.6(3)
C18	Ir1	N2	81.07(13)		C19	C18	Ir1	128.9(3)
C18	Ir1	C7	92.75(13)		C19	C18	C17	116.4(3)
C33	O1	Ir1	124.0(2)		C20	C19	C18	121.9(3)
C35	O2	Ir1	125.5(2)		C19	C20	C21	120.5(4)
C1	N1	Ir1	124.3(2)		C22	C21	C20	119.2(4)
C1	N1	C5	119.6(3)		C21	C22	C17	119.9(4)
C5	N1	Ir1	116.1(2)		C24	C23	C14	175.3(4)
C12	N2	Ir1	125.1(2)		C23	C24	C25	174.9(4)
C12	N2	C16	119.2(3)		C26	C25	C24	119.2(4)
C16	N2	Ir1	115.6(2)		C26	C25	C30	119.8(4)
N1	C1	C2	122.2(3)		C30	C25	C24	121.0(4)
C1	C2	C3	119.0(4)		C27	C26	C25	120.6(4)
C4	C3	C2	119.1(3)		C26	C27	C28	119.8(4)
C3	C4	C5	120.4(3)		C27	C28	C29	119.9(4)
N1	C5	C4	119.7(3)		C27	C28	C31	120.1(4)
N1	C5	C6	113.6(3)		C29	C28	C31	120.0(4)
C4	C5	C6	126.7(3)		C30	C29	C28	120.0(4)
C7	C6	C5	115.2(3)		C29	C30	C25	119.8(4)
C11	C6	C5	123.8(3)		N3	C31	C28	179.0(6)
C11	C6	C7	120.9(3)		O1	C33	C32	114.8(3)
C6	C7	Ir1	114.4(2)		O1	C33	C34	126.9(3)
C8	C7	Ir1	128.4(3)		C34	C33	C32	118.3(3)
C8	C7	C6	117.1(3)		C35	C34	C33	128.9(3)
C9	C8	C7	121.3(4)		O2	C35	C34	126.2(3)
C10	C9	C8	121.1(4)		O2	C35	C36	116.0(3)
C9	C10	C11	119.4(3)		C34	C35	C36	117.8(3)
C10	C11	C6	120.2(3)					

S4. Electrochemistry

Electrochemical analyses of the iridium complexes were carried out using an EmStat[2] potentiometer (PalmSens Instruments, West Lafayette, IN, USA), with platinum working, platinum counter and platinum pseudo-reference electrodes. Analyses were performed at a scan rate of 100 mV s^{-1}. The ferrocene/ferrocenium (Fc/Fc^+) or decamethylferrocene/decamethylferrocenium (Fc^*/Fc^{*+}) couple was used as the internal reference where the Fc/Fc^+ couple is at 0.0 V. Analytical grade solvents were used.

Table S4. Electrochemical data for complexes **1**–**7** in MeCN

**Complex**	**$E_{1/2}$ Ox (V_{Fc/Fc^+})**	**$E_{1/2}$ Red (V_{Fc/Fc^+})**	**HLG (eV)**
**1**[1]	0.39	-	
**2**	0.36	–2.33	2.69
**3**	0.40	–2.02	2.42
**4**	0.36[a]	-	
**5**	0.36	–2.23	2.59
**6**	0.39	–1.97	2.36
**7**	0.36	–2.23	2.59

[a] Recorded in DCM

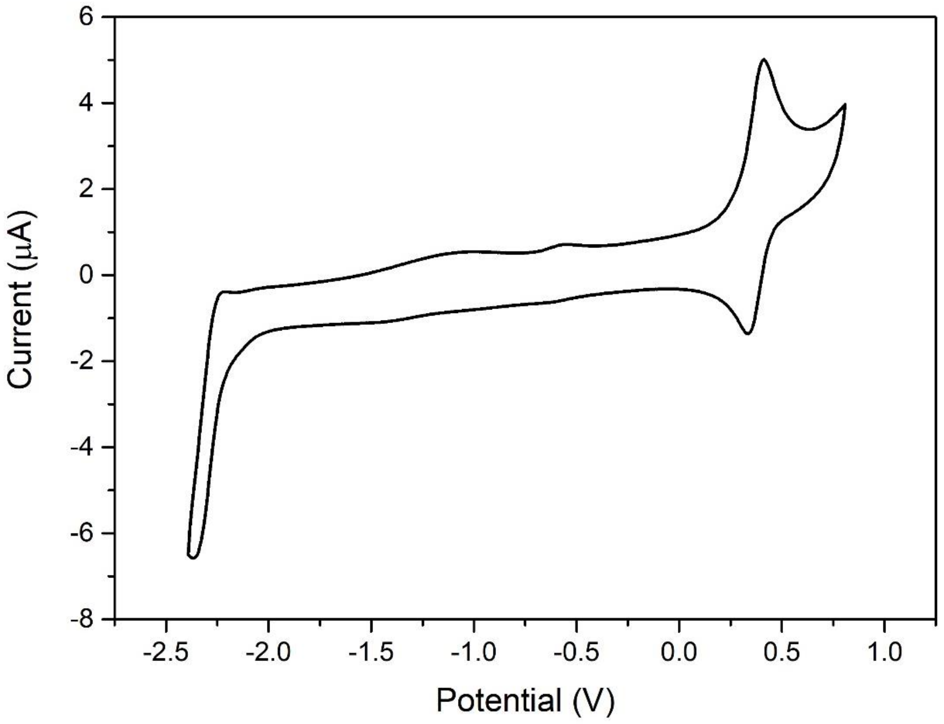

Figure S14. Cyclic voltammogram of complex **2** recorded with scan rate of 100 mV s^{-1}.

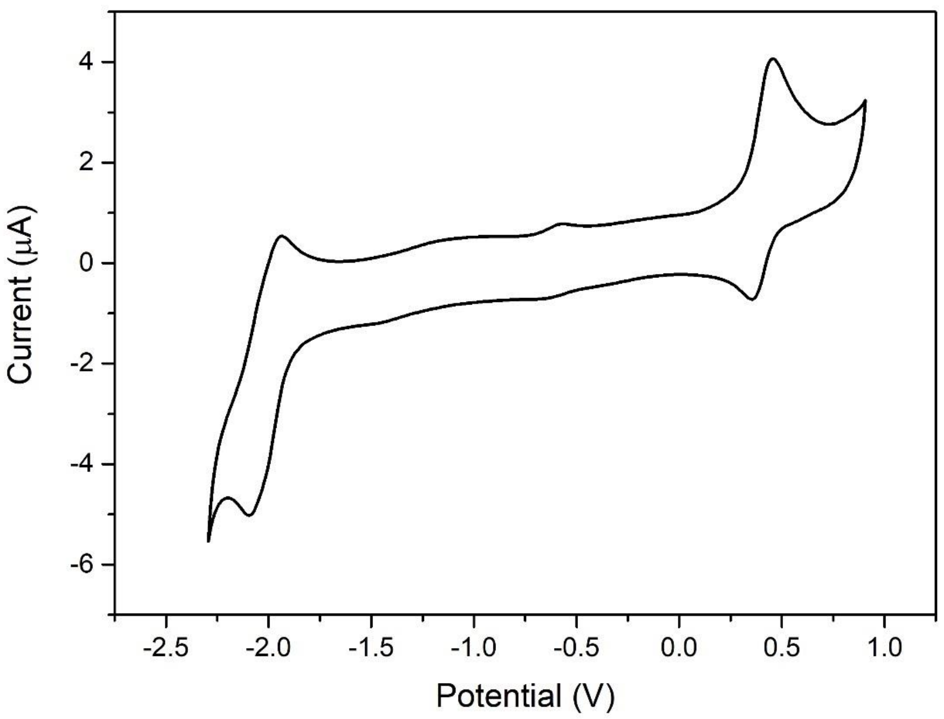

Figure S15. Cyclic voltammogram of complex **3** recorded with scan rate of 100 mV s^{-1}.

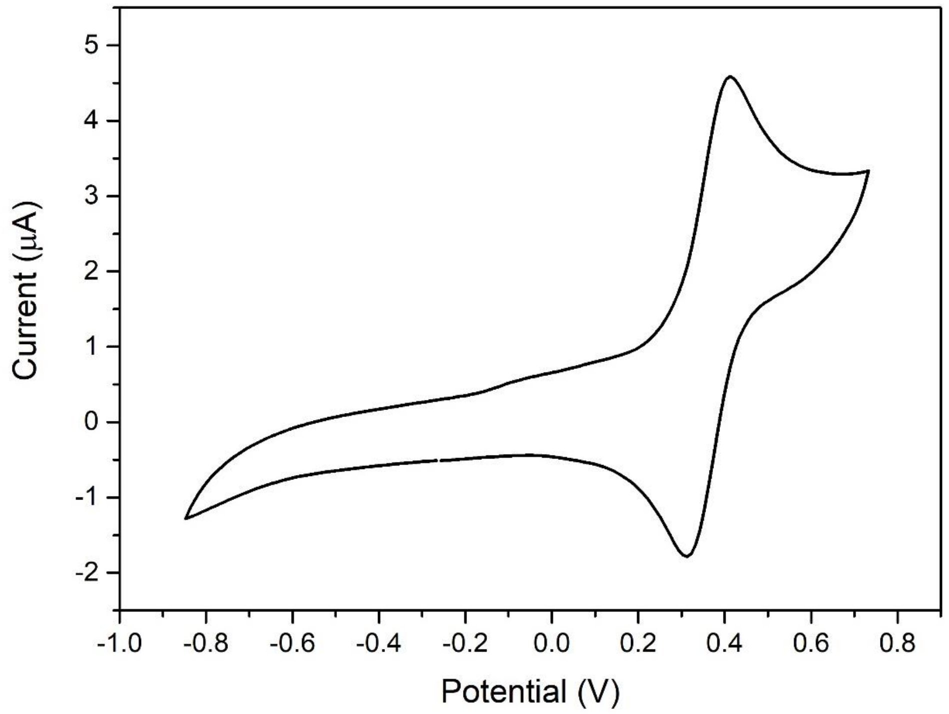

Figure S16. Cyclic voltammogram of complex **4** recorded with scan rate of 100 mV s^{-1}.

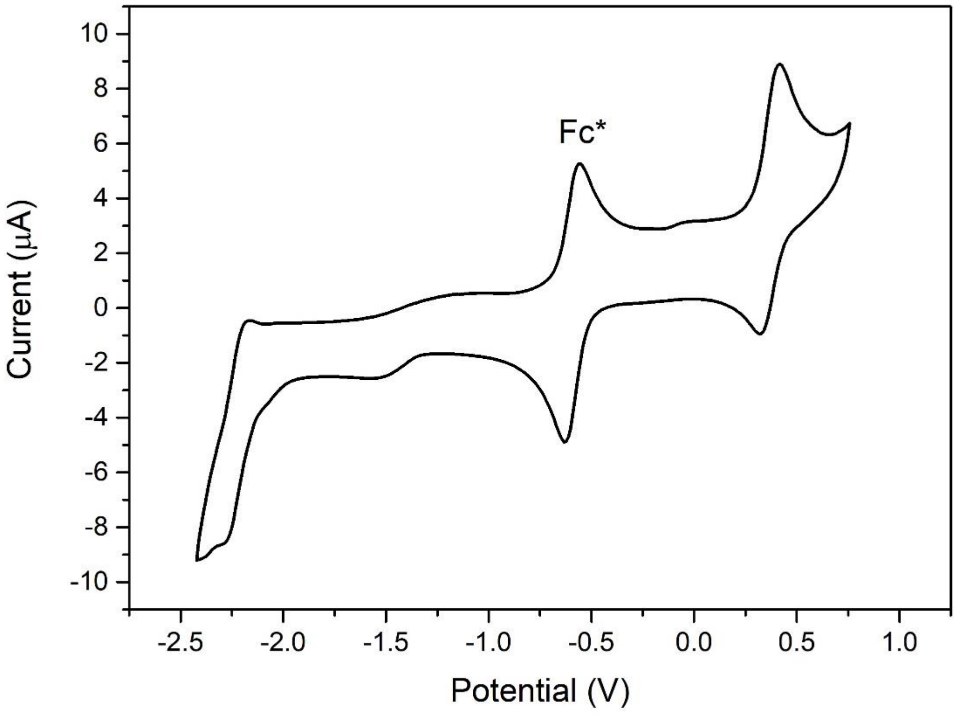

Figure S17. Cyclic voltammogram of complex **5** recorded with scan rate of 100 mV s^{-1}.

Note: the minor signal at ca. -1.5 V is attributed to the presence of trace oxygen.

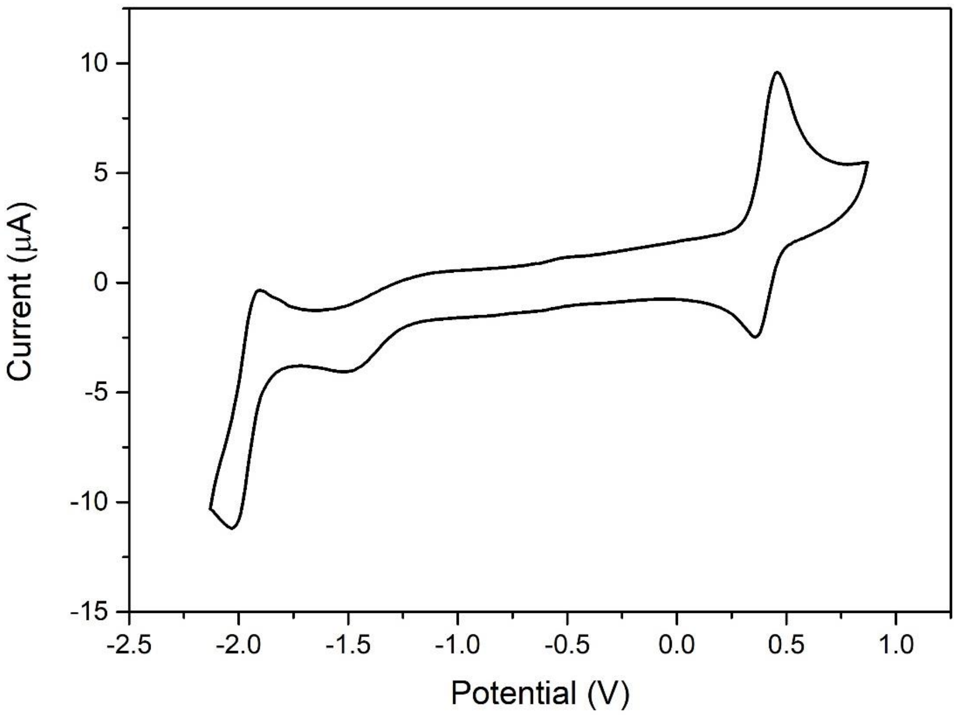

Figure S18. Cyclic voltammogram of complex **6** recorded with scan rate of 100 mV s^{-1}.

Note: the minor signal at ca. -1.5 V is attributed to the presence of trace oxygen.

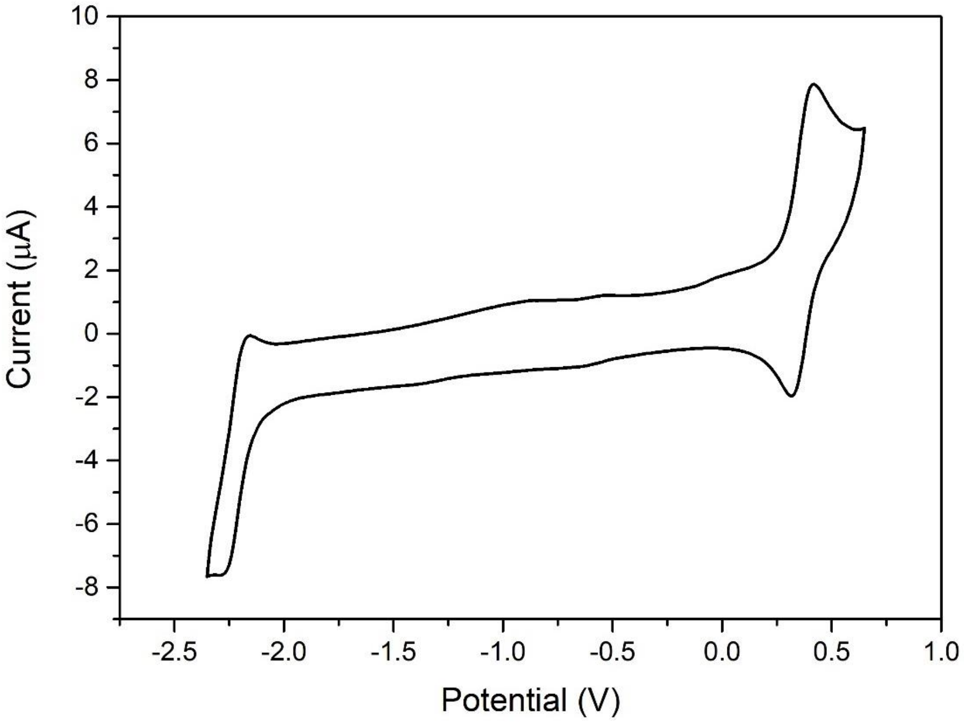

Figure S19. Cyclic voltammogram of complex **7** recorded with scan rate of 100 mV s^{-1}.

S5. Photophysical measurements

All photophysical measurements of iridium complexes (except solvatochromism) were performed using DCM as the solvent. The ultraviolet (UV)-visible spectra were measured on a UV2-100 spectrometer operated with Vison software (Unicam Digital, Leeds, UK). Samples were held in quartz cuvettes, with path length l = 1 cm. Excitation and emission photoluminescence spectra were recorded on a Spex® Fluorolog® 3-22 spectrofluorometer (Horiba Scientific, Irvine, CA, USA). Samples were degassed in quartz cuvettes of l = 1 cm fitted with Teflon J Young taps by repeated freeze–pump–thaw cycles using a turbomolecular pump until the pressure was stable. The solutions' absorbance was < 0.15 to minimize inner filter effects.

Photoluminescence quantum yields (PLQYs) were measured following our previously reported method (see below).[4] Quinine sulfate in 0.1 M H_2SO_4 (Φ_F: 0.546)[5] or rhodamine 101 in ethanol (Φ_F: 1.00)[6] as the references, the emission spectra of quinine sulfate were collected by exciting the samples at 360 nm. The PLQY of each complex was measured in duplicate and determined by the following method:

1. The UV-vis absorbance spectrum was recorded in quartz cuvette with path length l = 2 cm (to improve spectrum signal-to-noise), recording the absorbance at the excitation wavelength.
2. The same sample solution was transferred to the quartz fluorescence cell (a standard 1 cm cell modified with a Teflon Young's tap) degassed via repeated freeze-pump-thaw cycles before the emission spectrum was recorded.
3. The fully corrected fluorescence spectrum was integrated and the integrated intensity (the area of the fluorescence spectrum) was recorded.
4. Steps 1 to 3 were repeated for five additional solutions with increasing concentrations (with absorbance ranging from 0.02 to 0.1).
5. The integrated fluorescence intensity was plotted verse absorbance, which resulted a linear plot with gradient X ($Grad_X$).

6. Steps 1, 3, 4 and 5 were repeated for the chosen standard (quinine sulfate).
7. Fluorescence quantum yield for each complex was calculated using the following equation

$$\Phi_X = \Phi_{ST}\left(\frac{Grad_X}{Grad_{ST}}\right)\left(\frac{\eta^2{}_X}{\eta^2{}_{ST}}\right)$$

   Where ST and X denote standard and the measured complex, Φ is the fluorescence quantum yield, Grad the gradient from the plot of integrated fluorescence intensity vs. absorbance, and η the refractive index of the solvent.

Emission lifetimes were measured using a custom spectrometer by time-correlated single photon counting (TCSPC) using a pulsed diode laser (371 nm), made by IBH Ltd, running at 1 MHz. The fluorescence emission was collected at right angles to the excitation source. The emission wavelength was selected using a Horiba Jobin Yvon Triax 190 monochromator and was detected by using a cooled IBH TBX-04 photomultiplier (PMT). Timing was achieved using a 567 time-to-amplitude converter (Ortec, Melbourne, AU) and a pulse-height analyzer (PHA; E. G. & G. Trumpcard, Carlsbad, CA, USA). Data were recorded using Maestro (version 510) software. The data were transferred to a personal computer (PC) and analysed using non-linear regression to a single exponential decay, and the quality of fit was established by reduced χ^2 and random residuals. The decay data were fitted to a single exponential function.

Electronic absorption spectra

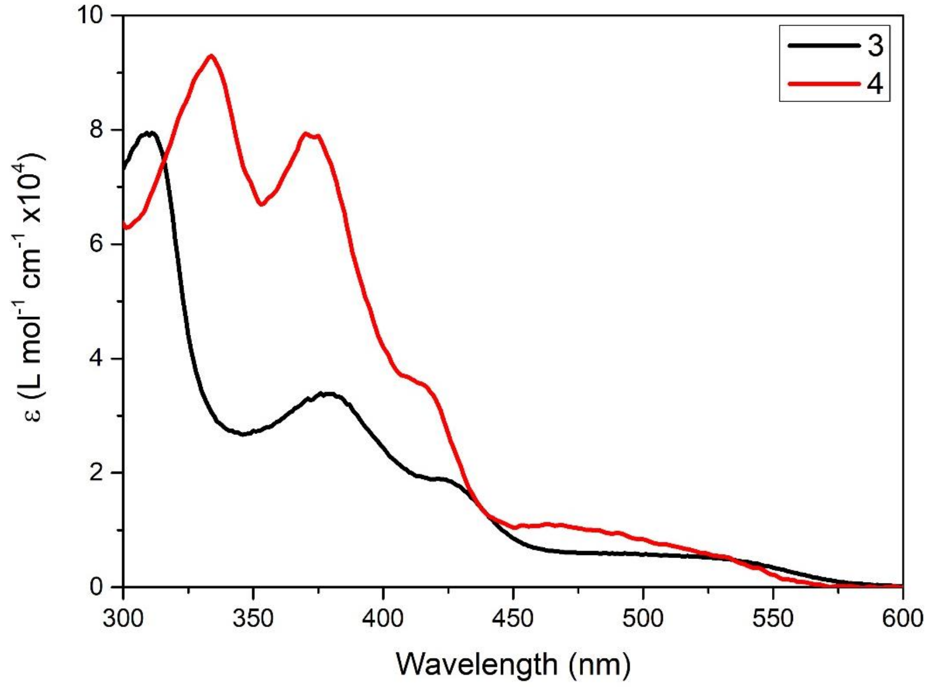

Figure S20. Electronic absorption spectra of complexes **3** and **4** recorded in DCM solution.

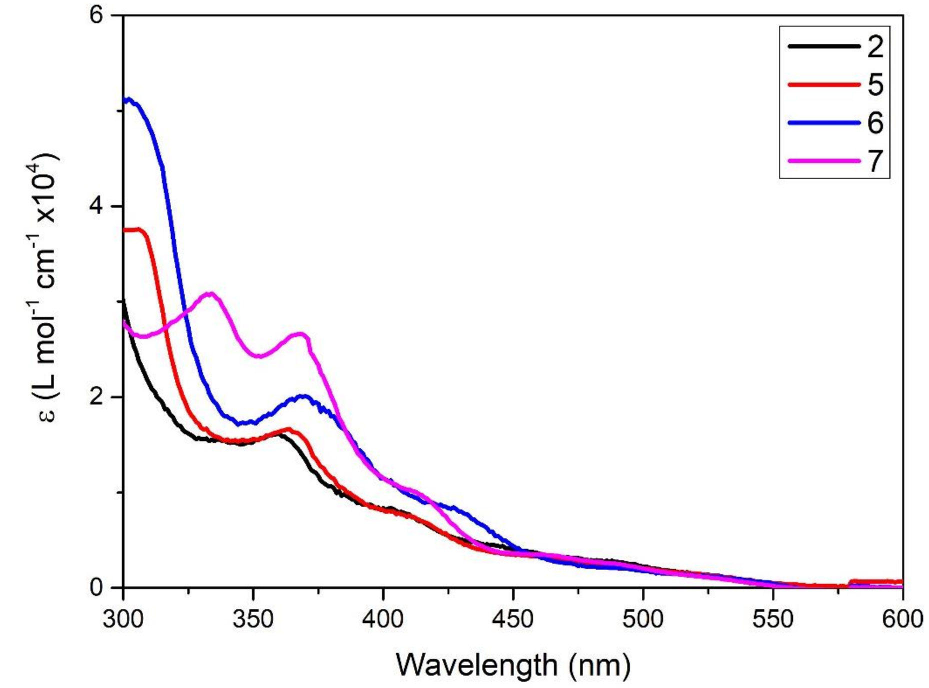

Figure S21. Electronic absorbance spectra of complexes **2**, **5**, **6**, and **7** recorded in DCM solution.

Photostability

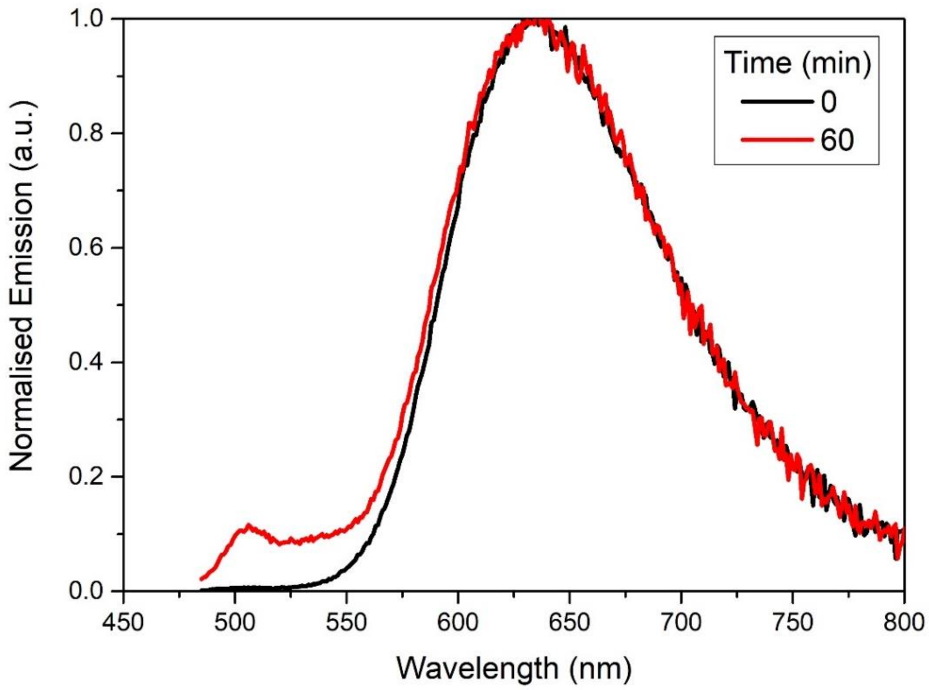

Figure S22. Photostability of complex **3** recorded in DCM solution.

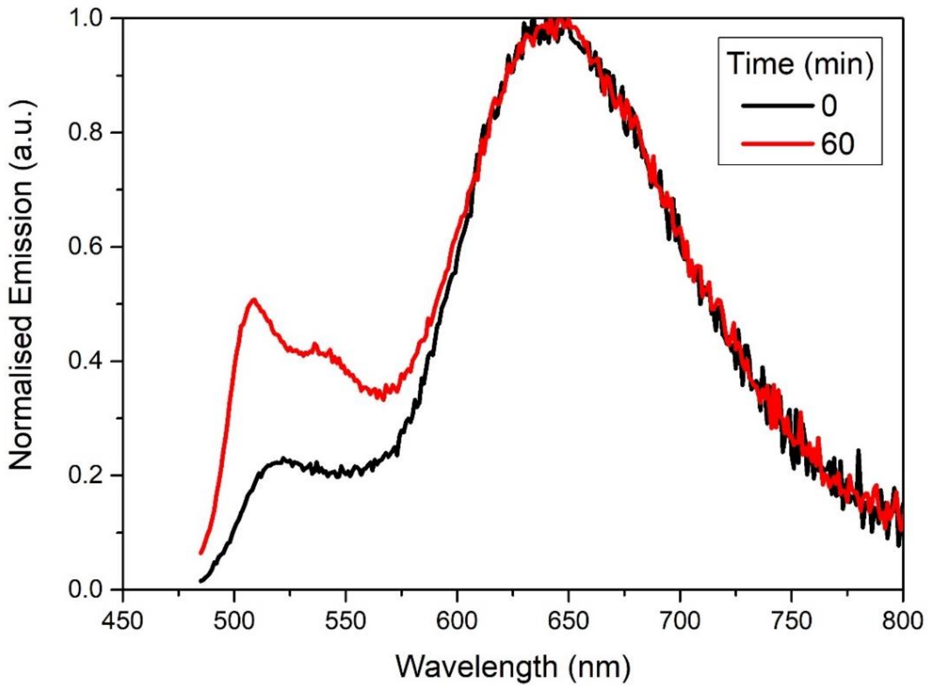

Figure S23. Photostability of complex **6** recorded in DCM solution.

Solvatochromism

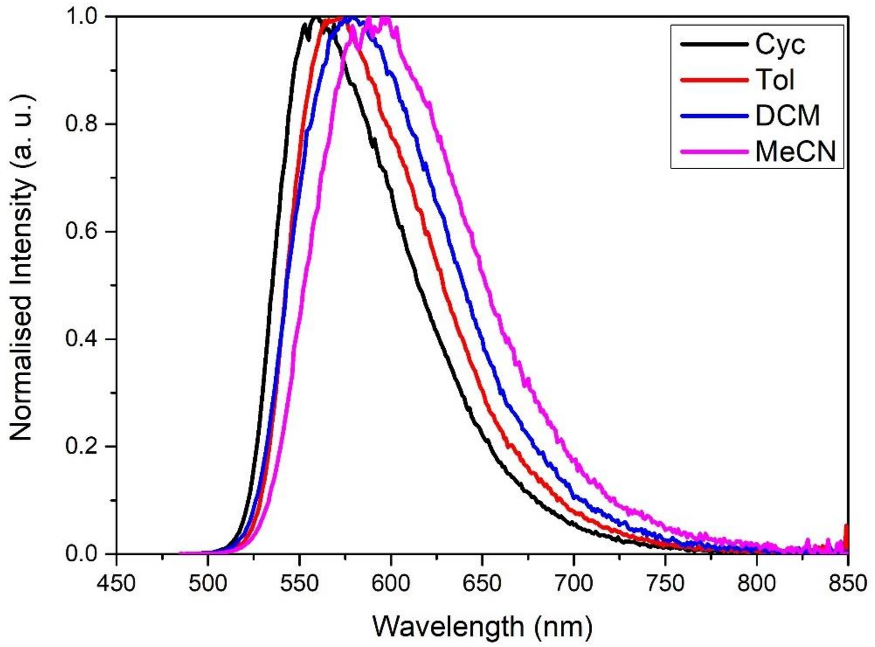

Figure S24. Emission of complex **1** recorded in cyclohexane (Cyc), toluene (Tol), dichloromethane (DCM), and acetonitrile (MeCN).

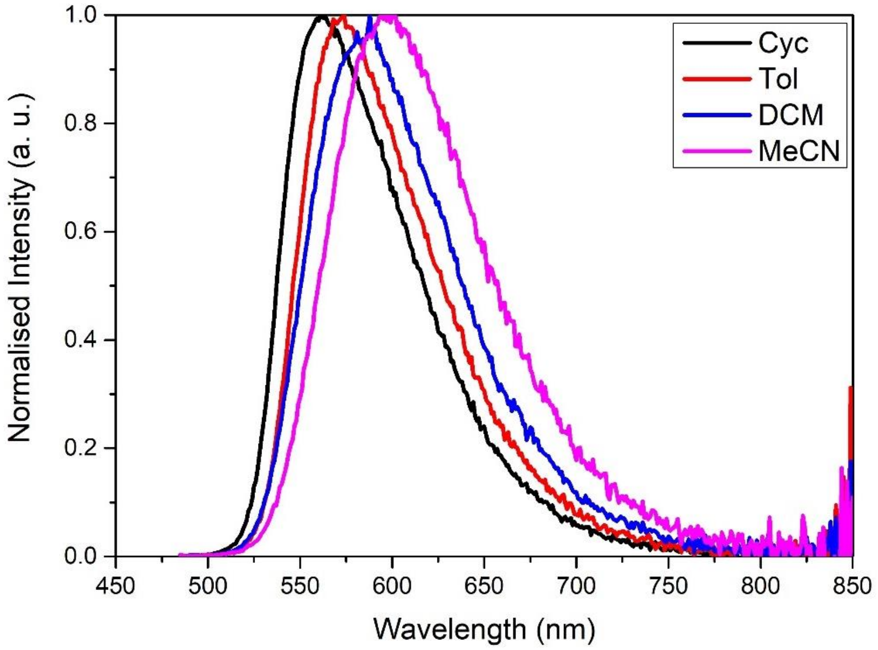

Figure S25. Emission of complex **2** recorded in cyclohexane (Cyc), toluene (Tol), dichloromethane (DCM), and acetonitrile (MeCN).

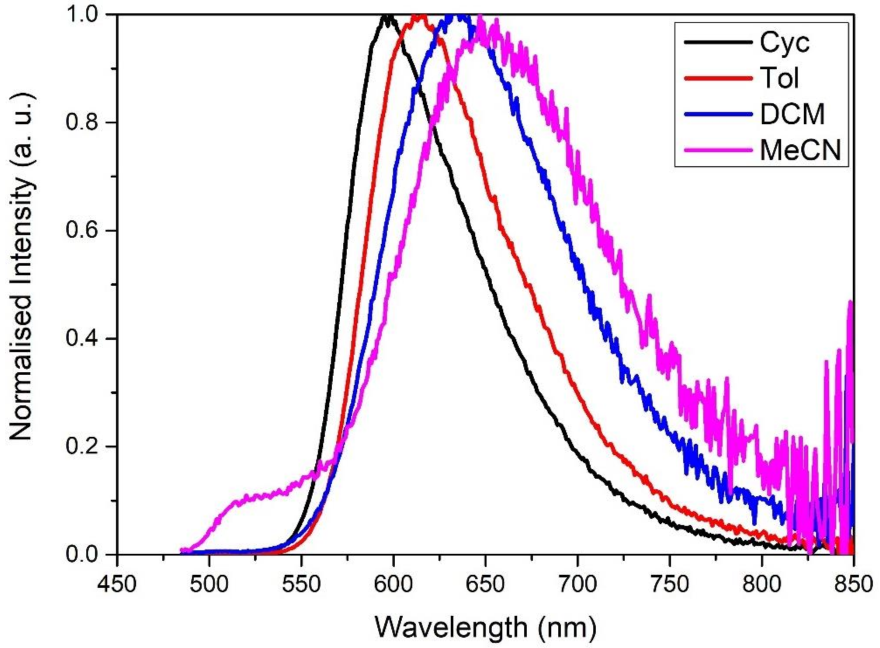

Figure S26. Emission of complex **3** recorded in cyclohexane (Cyc), toluene (Tol), dichloromethane (DCM), and acetonitrile (MeCN).

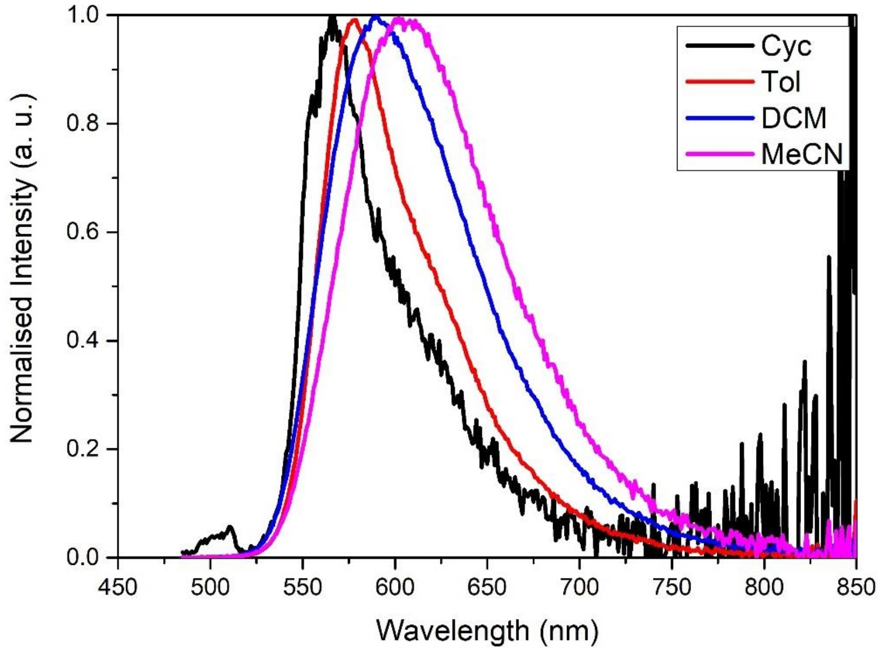

Figure S27. Emission of complex **4** recorded in cyclohexane (Cyc), toluene (Tol), dichloromethane (DCM), and acetonitrile (MeCN).

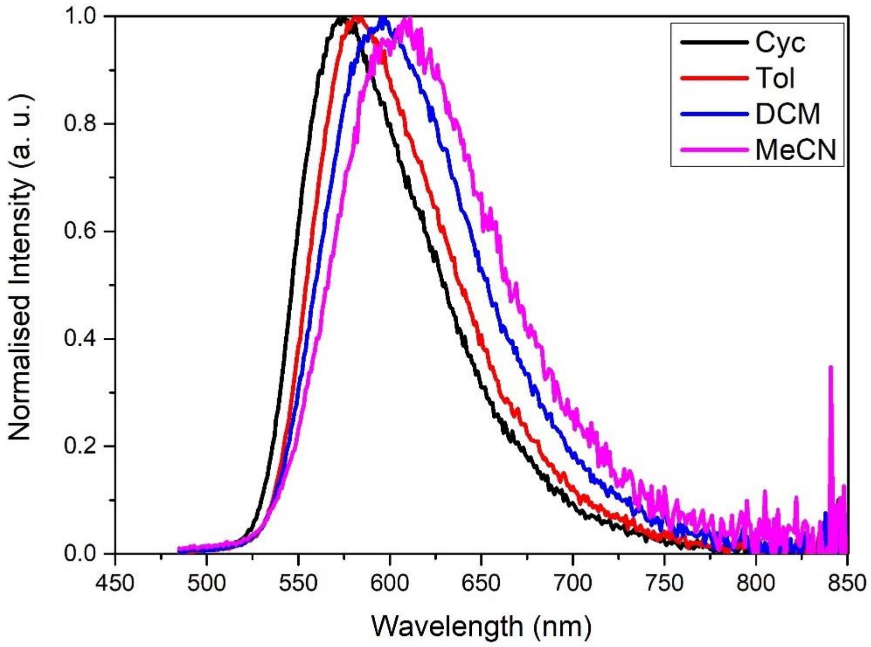

Figure S28. Emission of complex **5** recorded in cyclohexane (Cyc), toluene (Tol), dichloromethane (DCM), and acetonitrile (MeCN).

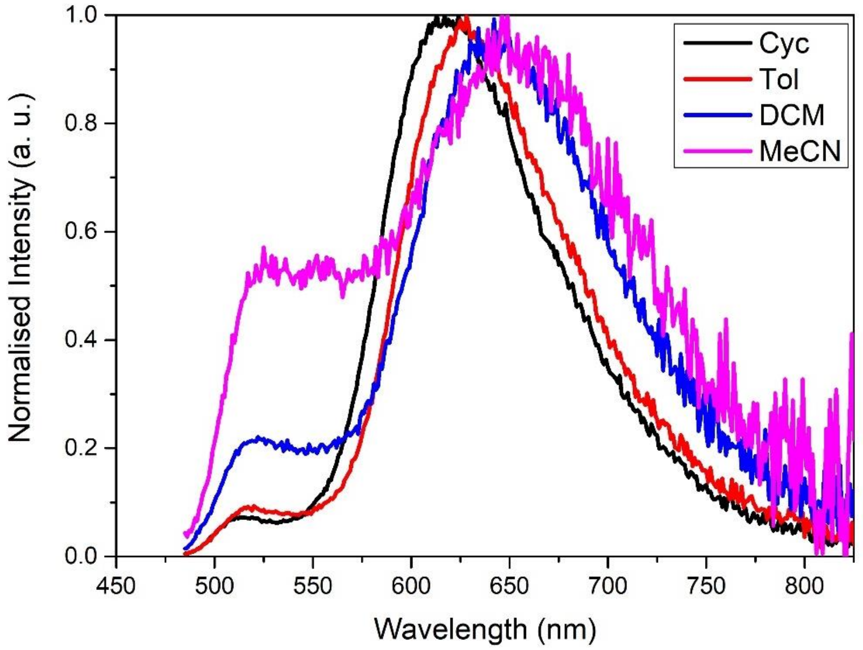

Figure S29. Emission of complex **6** recorded in cyclohexane (Cyc), toluene (Tol), dichloromethane (DCM), and acetonitrile (MeCN).

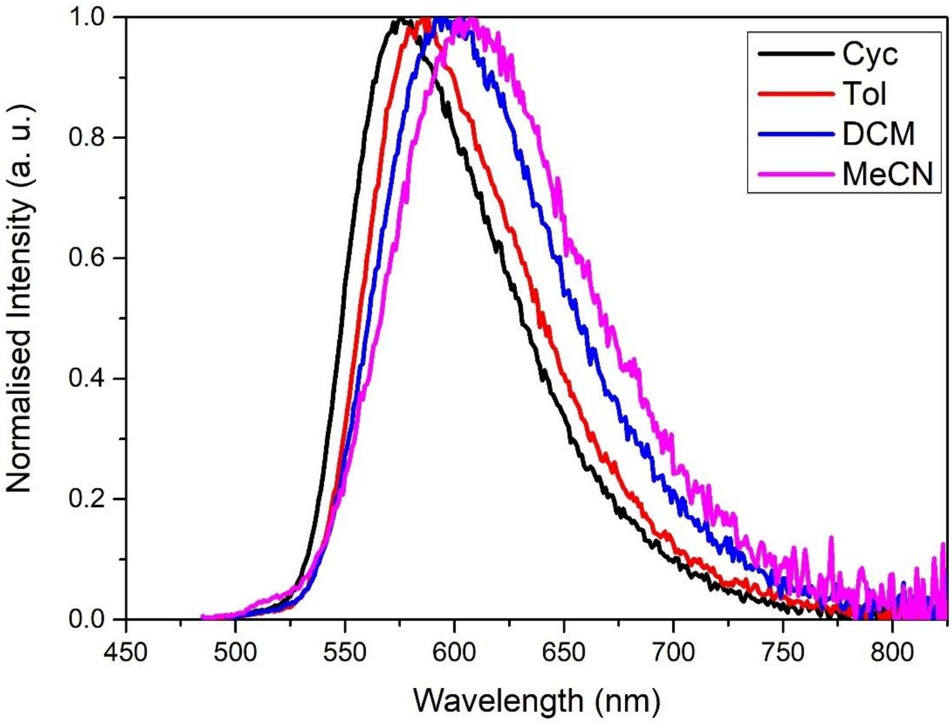

Figure S30. Emission of complex **7** recorded in cyclohexane (Cyc), toluene (Tol), dichloromethane (DCM), and acetonitrile (MeCN).

Lifetime Traces

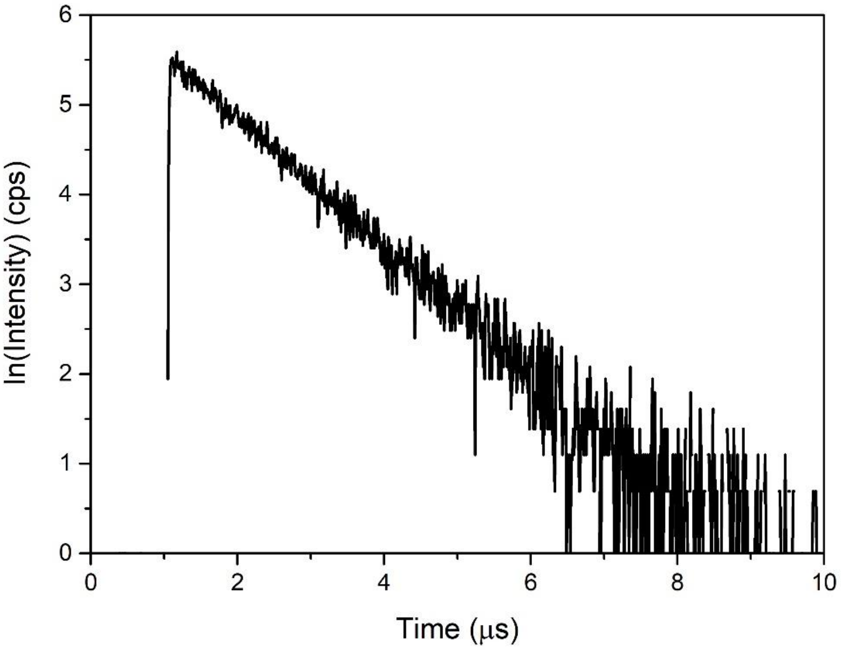

Figure S31. Lifetime trace for complex **2** recorded in DCM excited at 488 nm.

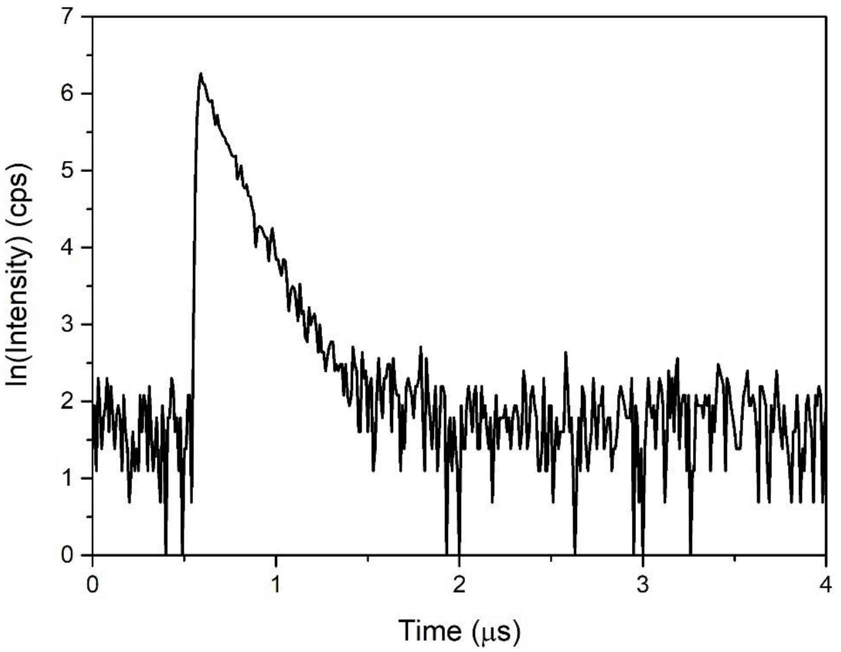

Figure S32. Lifetime trace for complex **3** recorded in DCM excited at 488 nm.

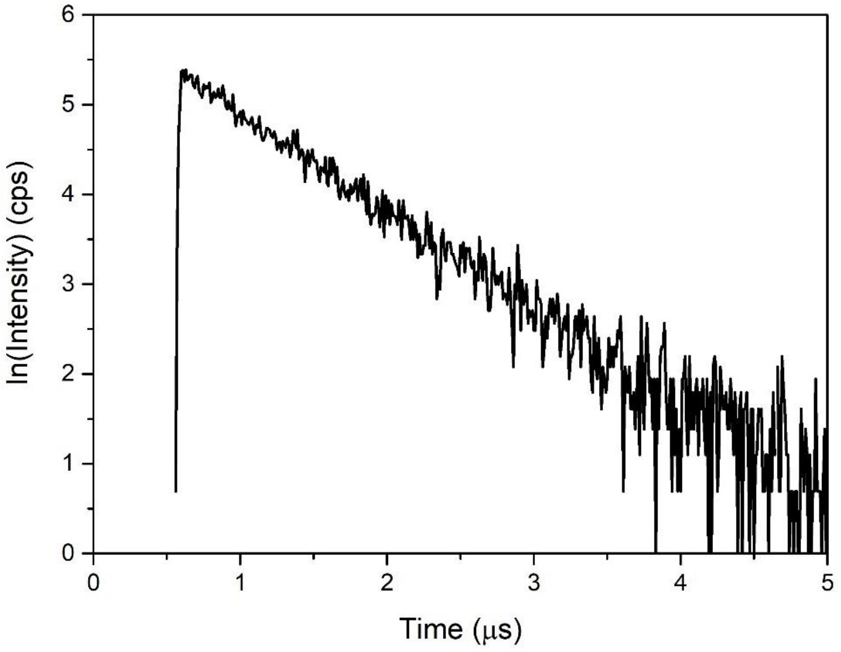

Figure S33. Lifetime trace for complex **4** recorded in DCM excited at 488 nm.

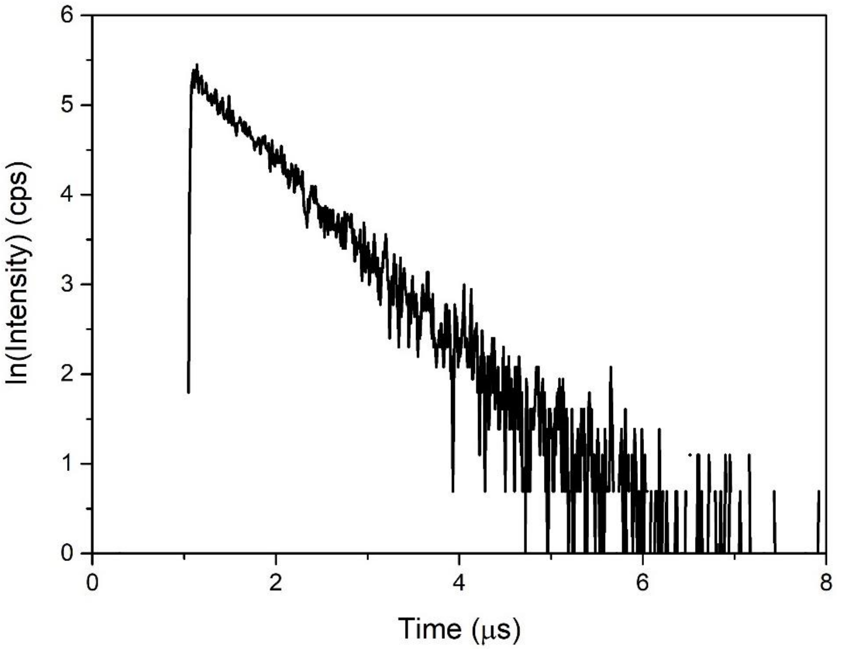

Figure S34. Lifetime trace for complex **5** recorded in DCM excited at 488 nm.

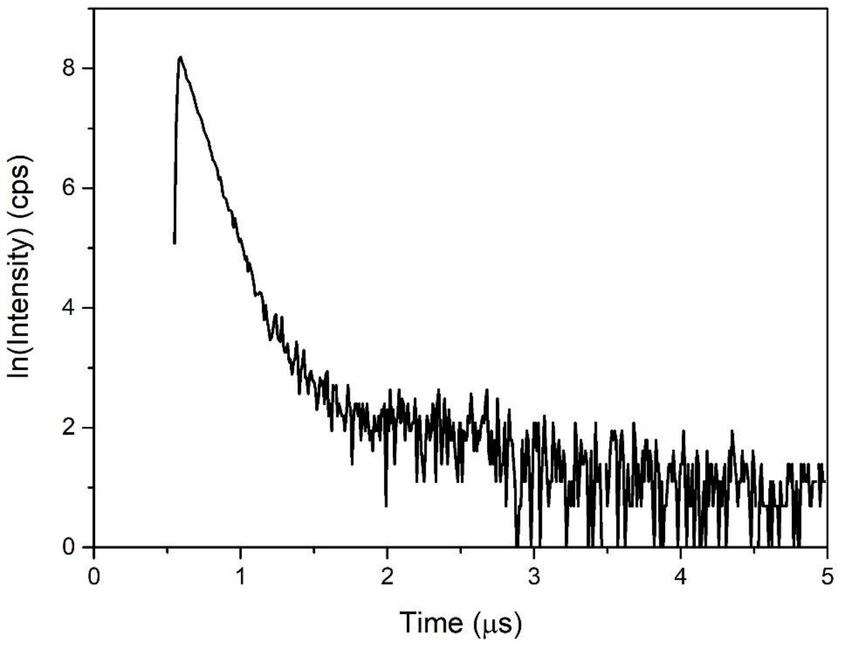

Figure S35. Lifetime trace for complex **6** recorded in DCM excited at 488 nm.

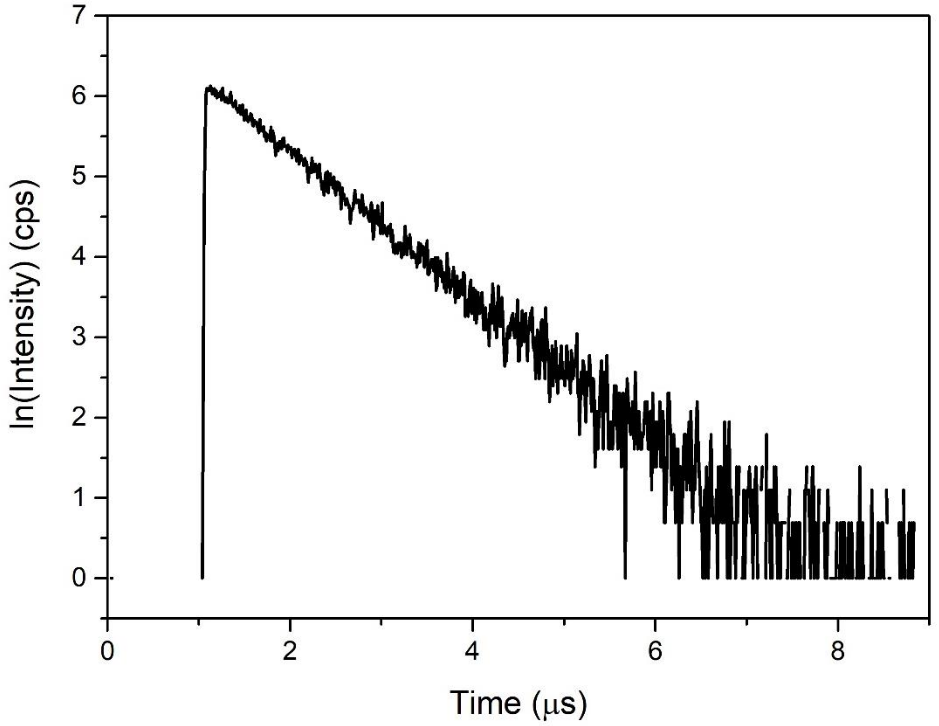

Figure S36. Lifetime trace for complex **7** recorded in DCM excited at 488 nm.

PLQY Measurements

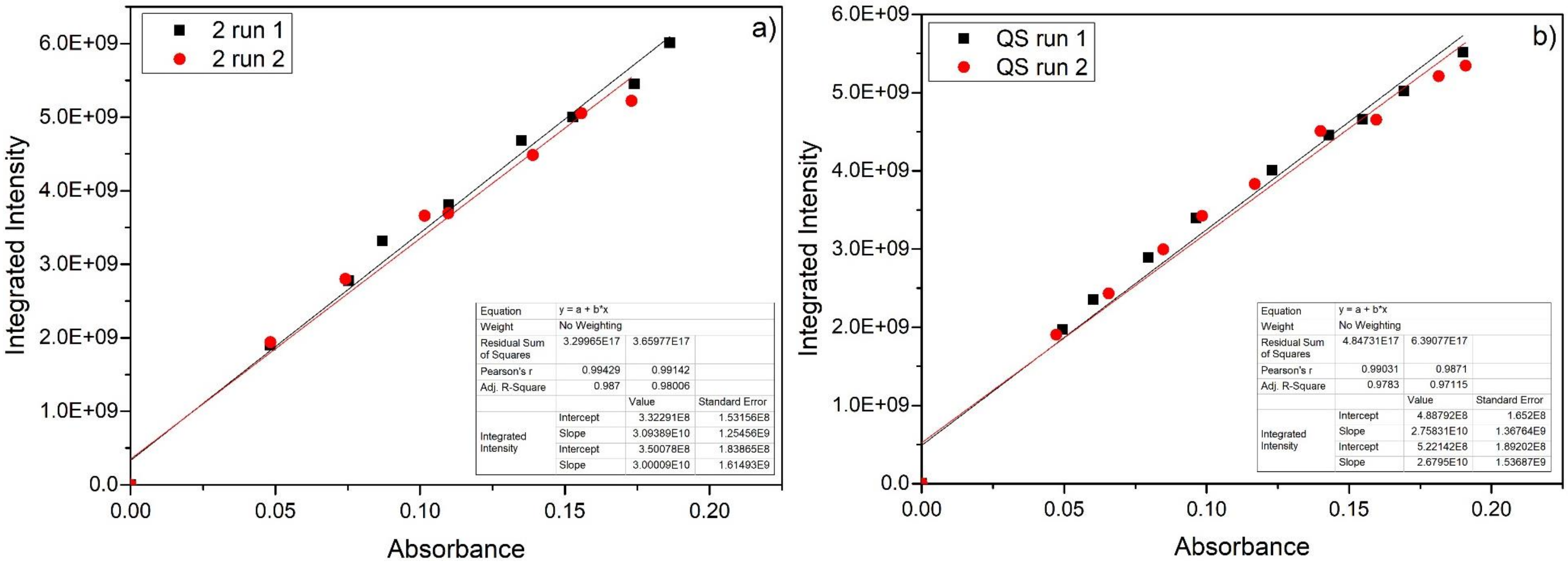

Figure S37. Integrated Intensity vs. Absorbance for a) **2** and b) quinine sulfate.

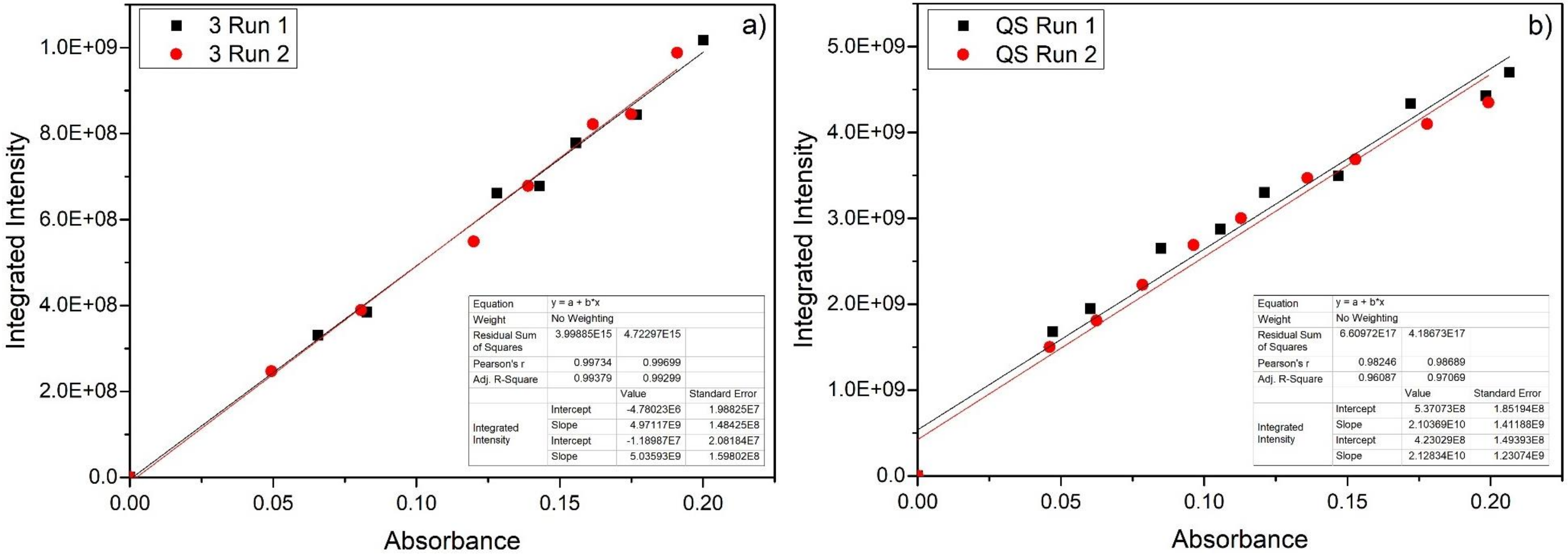

Figure S38. Integrated Intensity vs. Absorbance for a) **3** and b) quinine sulfate.

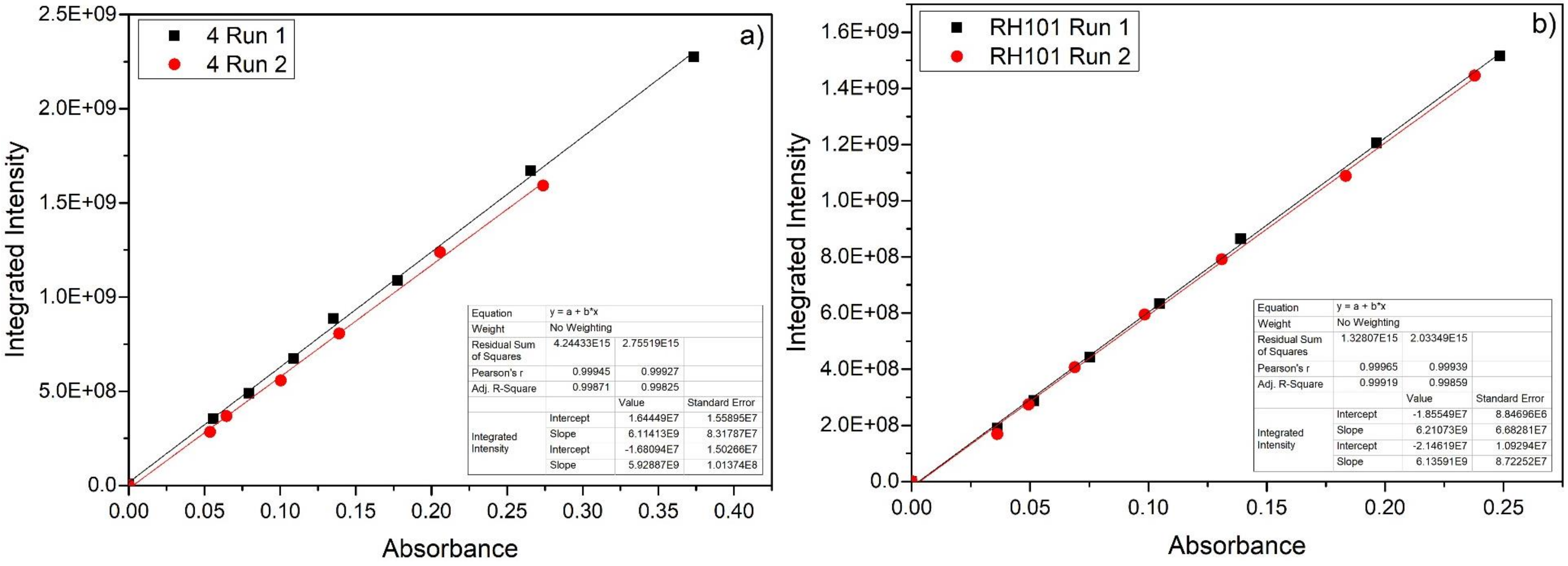

Figure S39. Integrated Intensity vs. Absorbance for a) **4** and b) rhodamine 101.

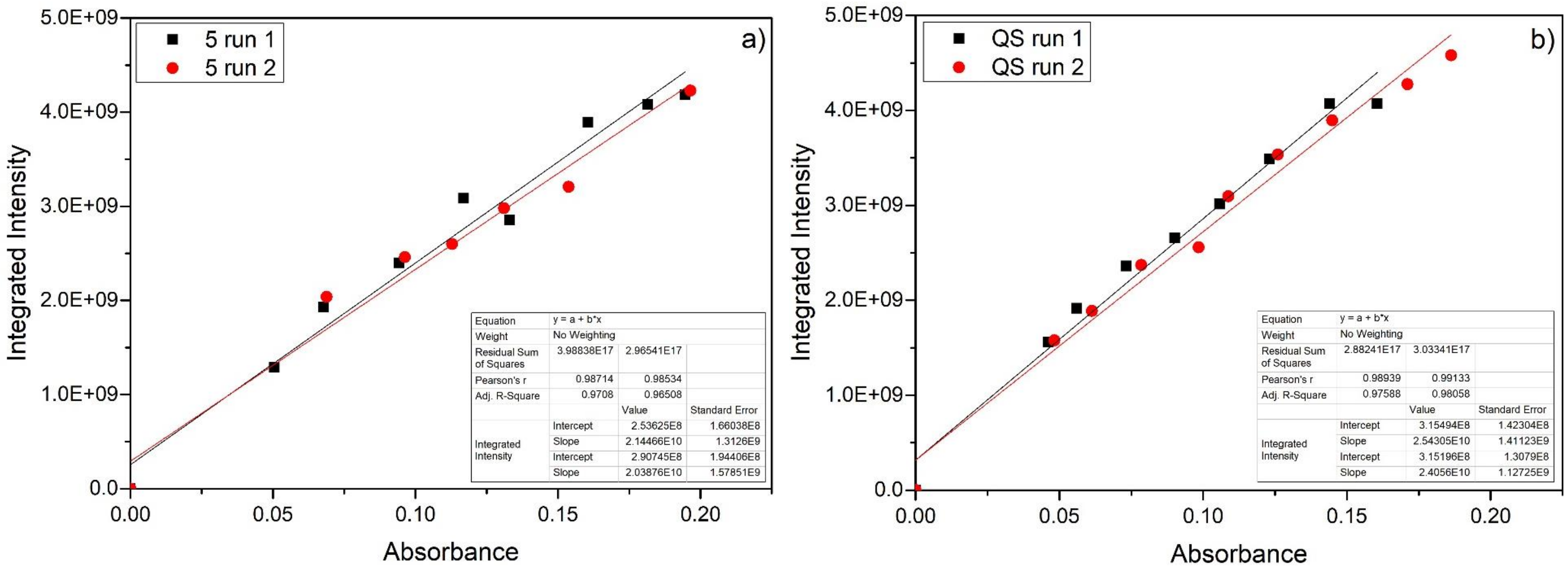

Figure S40. Integrated Intensity vs. Absorbance for a) **5** and b) quinine sulfate.

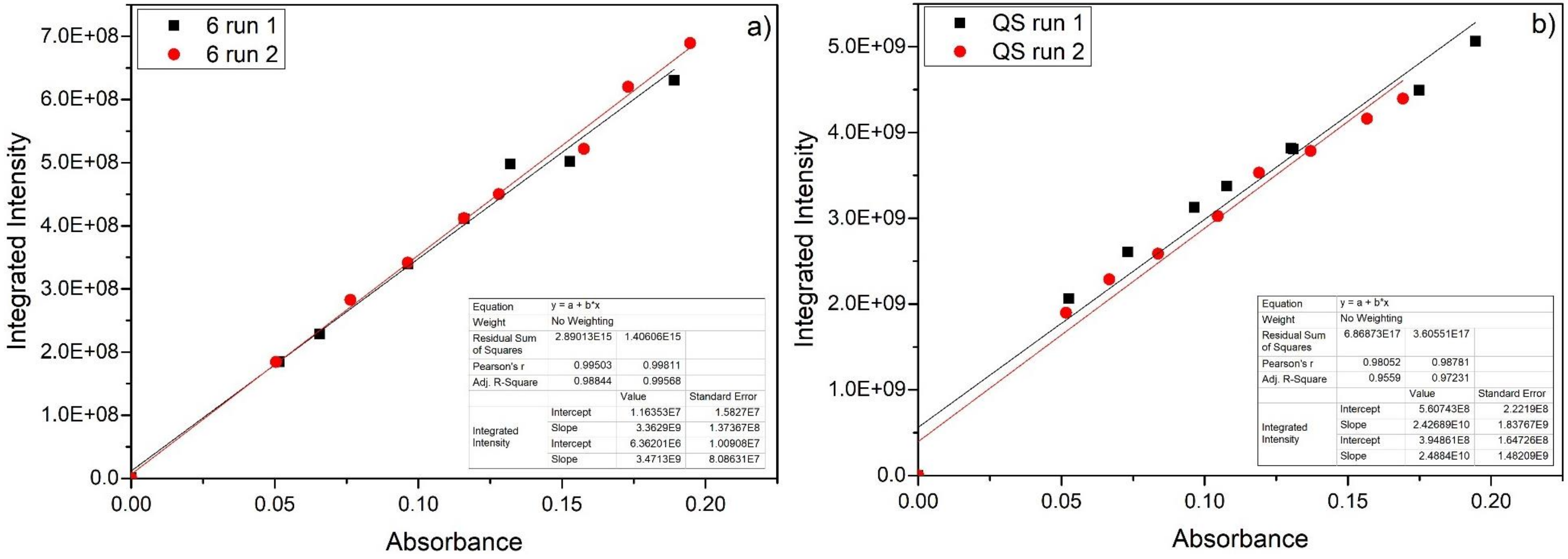

Figure S41. Integrated Intensity vs. Absorbance for a) **6** and b) quinine sulfate.

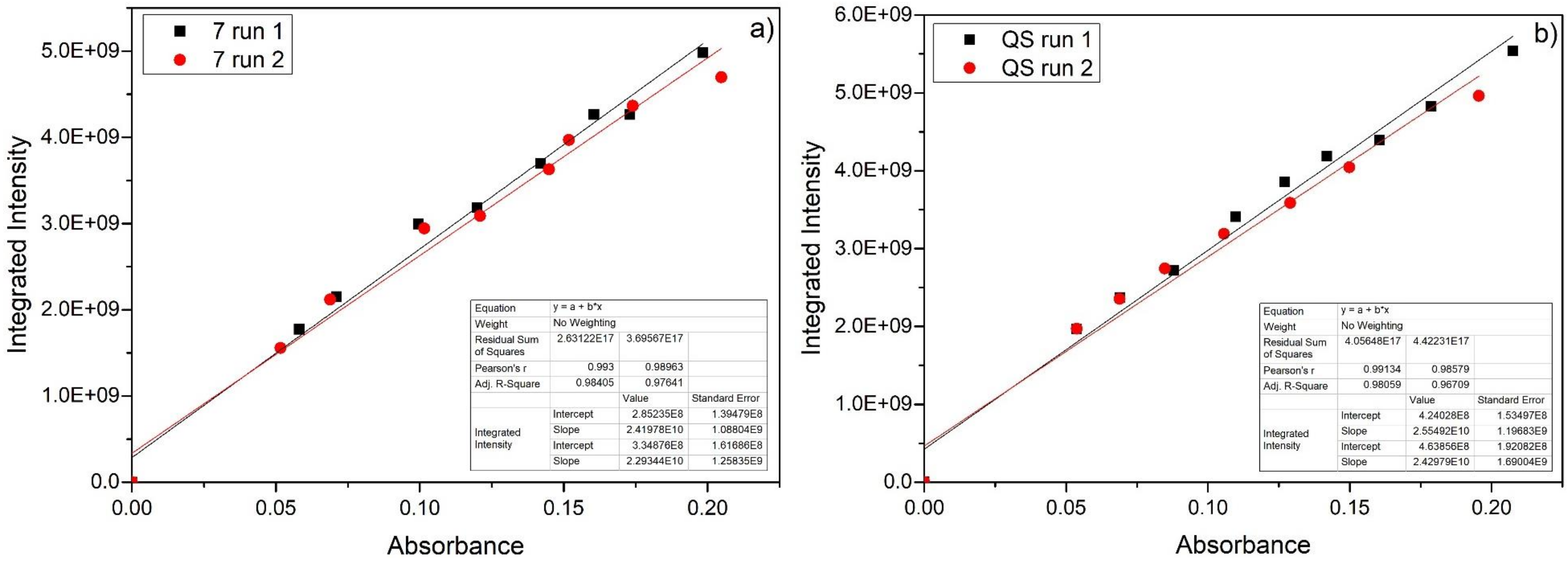

Figure S42. Integrated Intensity vs. Absorbance for a) **7** and b) quinine sulfate.

S6. Calculations

Hybrid density functional theory (DFT) calculations were performed using the Gaussian16 package (Gaussian, Inc) [7] All calculations used the B3LYP[8] functional with a mixed basis set LANL2DZ/3-21G* where the pseudopotential LANL2DZ[9, 10] was applied to iridium and the 3-21G* basis set[11, 12] for all other atoms. The fully optimised geometries were confirmed as true minima with no imaginary frequencies found from frequency calculations. The rotational barriers were estimated from constrained geometries with fixed torsional angles so that the two rings attached to the ethynyl bridge are perpendicular to each other. A frequency calculation on each constrained geometry showed one imaginary frequency indicating a rotation barrier transition state. Orbital figures were drawn using GabEdit[13] and the group contributions to each orbital were estimated as a percentage (%) with GaussSum[14].

Table S5. Comparison of calculated and experimental bond lengths in angstroms for fully optimised and X-ray geometries of **6**.

Bond length	Calculated	Experimental	Difference
Ir-O	2.168	2.164(2)	0.004
Ir-O	2.168	2.142(2)	0.026
Ir-N	2.051	2.034(3)	0.017
Ir-N	2.041	2.024(3)	0.017
Ir-C	2.011	1.993(3)	0.018
Ir-C	2.009	1.981(4)	0.028

Table S6. Total and relative energy data

Complex	Electronic Energy (au)	Relative Electronic Energy (kJ mol^{-1})	Gibbs Free Energy (au)	Relative Gibbs Free Energy (kJ mol^{-1})
**1**	-2834.195469		-2833.287705	
**2**	-2117.296306		-2116.652848	
**3**	-2194.899904	0.00	-2194.36803	0.00
**3** (90°,90°)	-2194.896722	8.37	-2194.360057	20.92
**4**	-2882.141402	0.00	-2881.55536	0.00
**4** (90°,90°)	-2882.138160	8.49	-2881.547061	21.80
**5**	-1705.917279	0.00	-1705.457135	0.00
**5** (90°)	-1705.915704	4.14	-1705.453432	9.71
**6**	-1797.647989	0.00	-1797.191808	0.00
**6** (90°)	-1797.646393	4.18	-1797.188056	9.83
**7**	-2141.268611	0.00	-2140.785541	0.00
**7** (90°)	-2141.267023	4.18	-2140.781504	10.59

Electronic structure data

Table S7. Comparison of calculated HOMO and LUMO energies in eV with observed electrochemical data.

Complex	Calculated HOMO	Calculated LUMO	Calculated HLG	Observed HOMO[a]	Observed LUMO[a]	Observed HLG[a]
**1**	-4.79	-1.73	3.06	-4.79	-	-
**2**	-4.77	-1.70	3.07	-4.76	-2.07	2.69
**3**<br>**3** (90°,90°)	-5.05<br>-5.04	-2.53<br>-2.03	2.52<br>3.01	-4.80	-2.38	2.42
**4**<br>**4** (90°,90°)	-4.86<br>-4.87	-2.10<br>-1.77	2.76<br>3.10	-4.76	-	-
**5**<br>**5** (90°)	-4.77<br>-4.78	-1.91<br>-1.66	2.86<br>3.12	-4.76	-2.17	2.59
**6**<br>**6** (90°)	-4.91<br>-4.90	-2.44<br>-1.97	2.47<br>2.93	-4.79	-2.43	2.36
**7**<br>**7** (90°)	-4.81<br>-4.81	-2.06<br>-1.72	2.75<br>3.09	-4.76	-2.17	2.59

[a] Observed potential values in V converted to E(HOMO/LUMO) = – 4.4 – $E_{1/2}$(Ox/Red) V for comparison with calculated E(HOMO/LUMO) values.

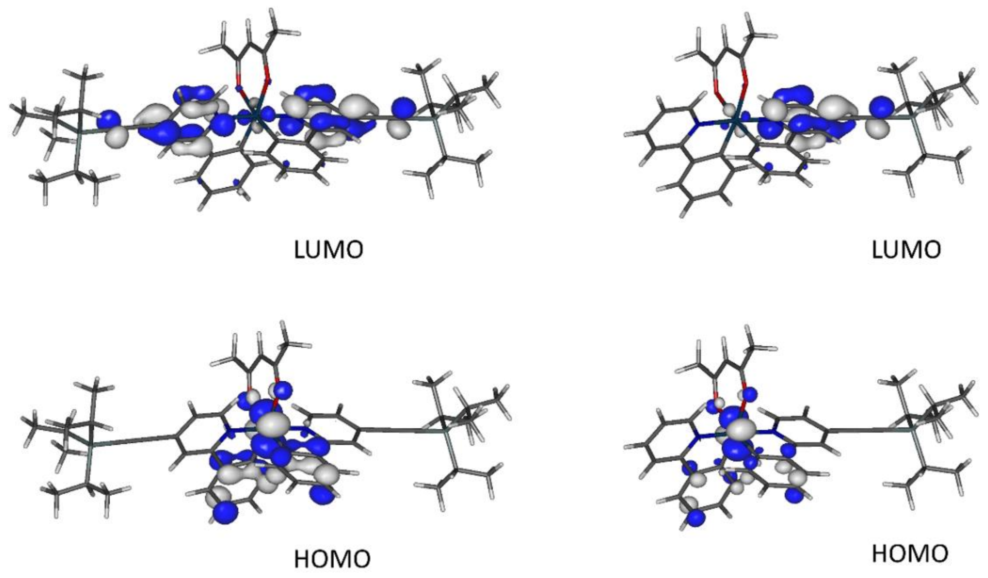

1 **2**

Figure S43. Frontier molecular orbitals for **1** and **2**. Isocontours drawn at 0.04 and 0.055 e bohr $^{-3/2}$ for **1** and **2** respectively.

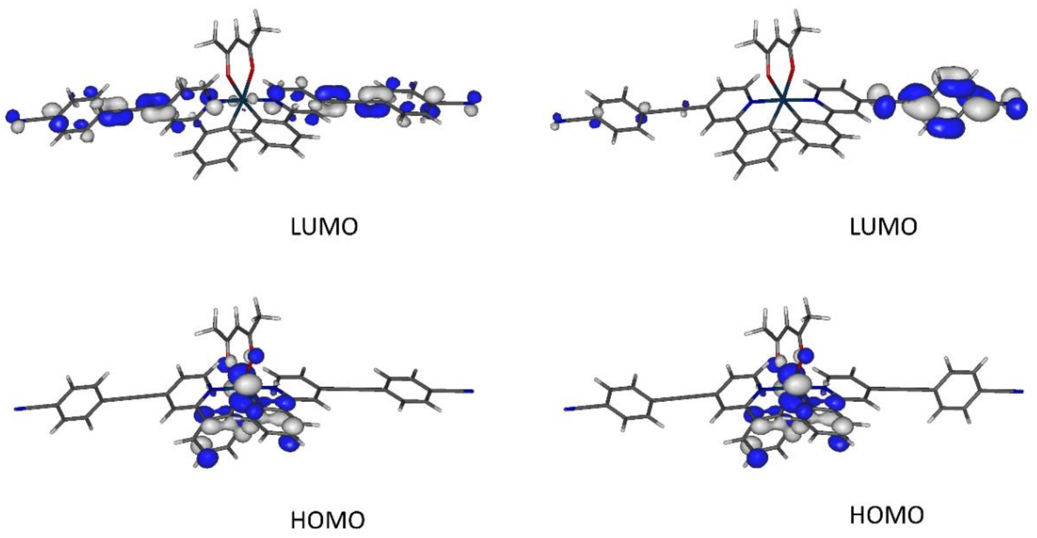

3 **3** (90°,90°)

Figure S44. Frontier molecular orbitals for the fully optimised geometry **3** where the phenylene rings attached to the ethynyl bridge are coplanar to each other and the constrained geometry **3** (90°, 90°) where the phenylene rings attached to the ethynyl bridge are constrained to be perpendicular to each other. Isocontours are at 0.04 e bohr $^{-3/2}$.

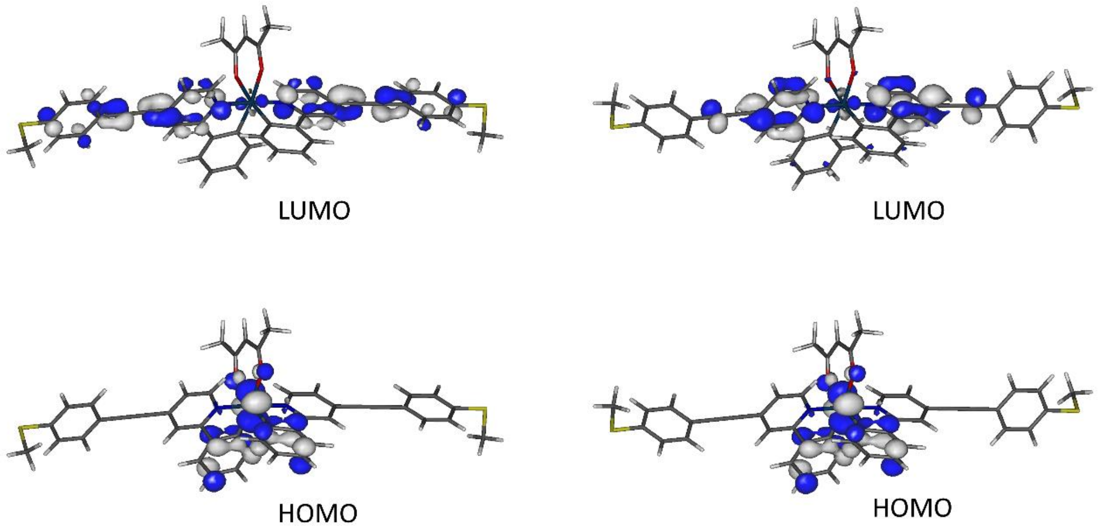

4 **4** (90°, 90°)

Figure S45. Frontier molecular orbitals for the fully optimised geometry **4** and the constrained geometry **4** (90°, 90°). Isocontours at 0.04 e bohr $^{-3/2}$.

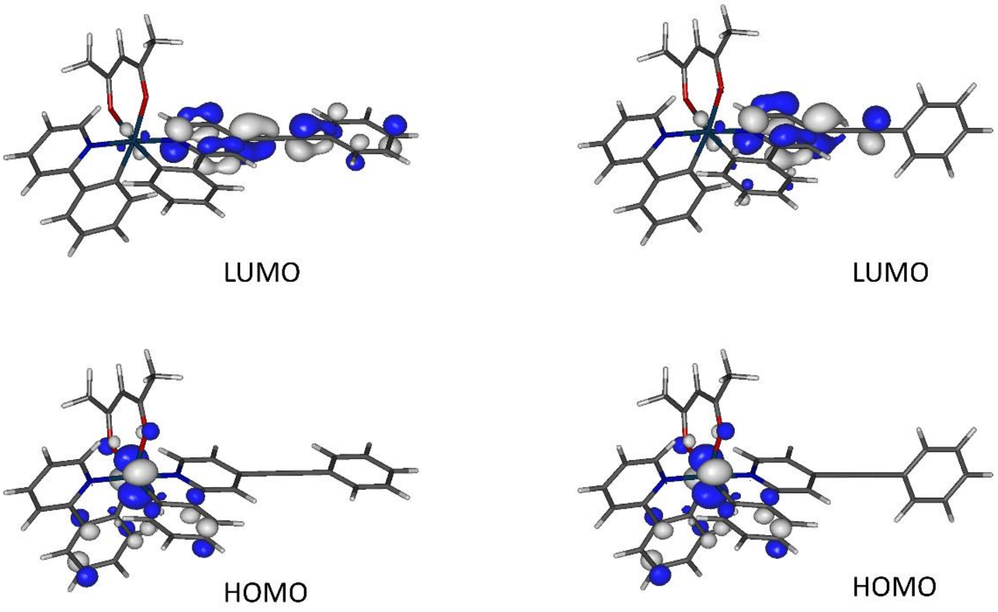

5 **5** (90°)

Figure S46. Frontier molecular orbitals for the fully optimised geometry **5** and the constrained geometry **5** (90°). Isocontours at 0.055 e bohr $^{-3/2}$.

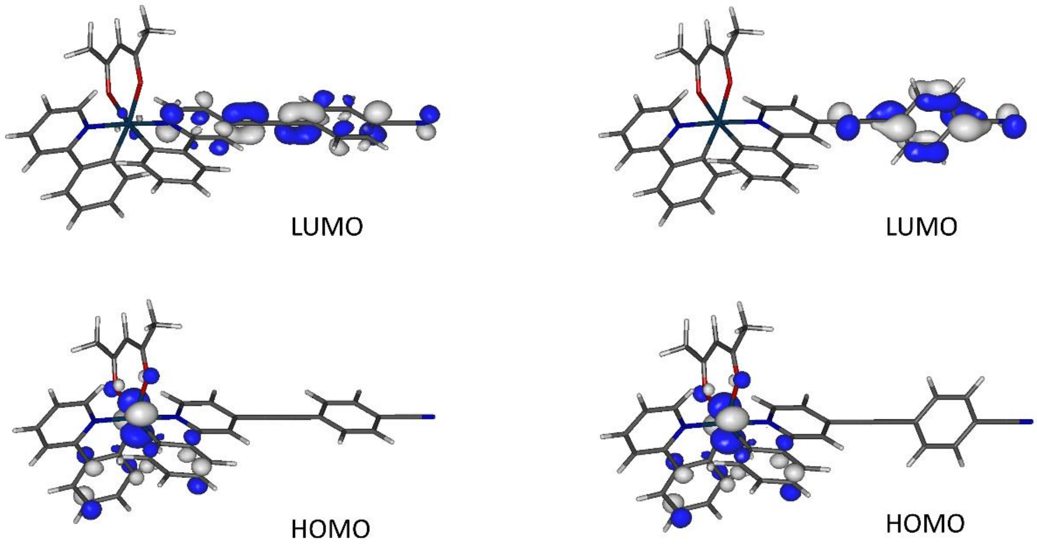

6 **6** (90°)

Figure S47. Frontier molecular orbitals for the fully optimised geometry **6** and the constrained geometry **6** (90°). Isocontours at 0.055 e bohr $^{-3/2}$.

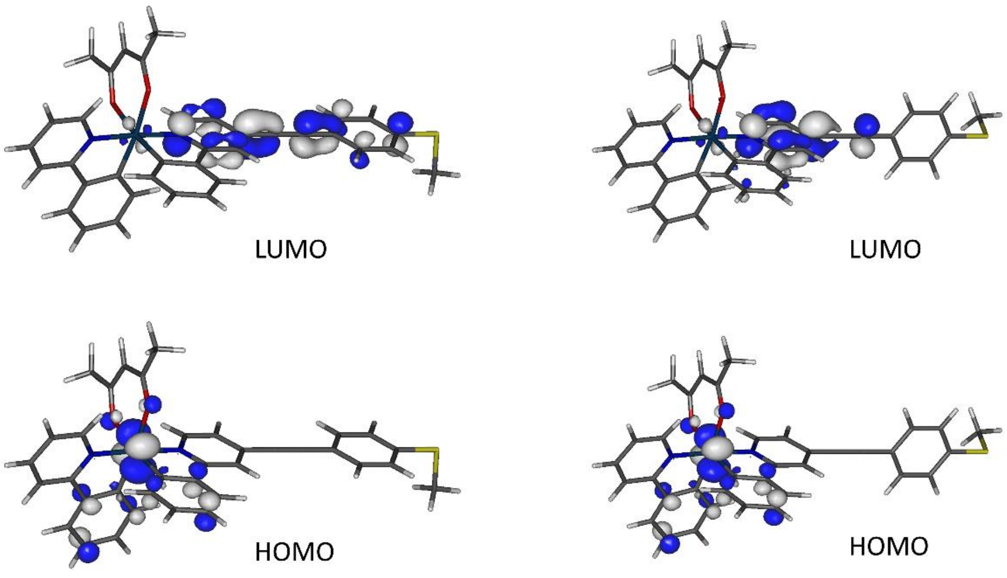

7 **7** (90°)

Figure S48. Frontier molecular orbitals for the fully optimised geometry **7** and the constrained geometry **7** (90°). Isocontours at 0.055 e bohr $^{-3/2}$.

Time-dependent density functional theory (TD-DFT) data

Table S8. Comparison of calculated $S_0 \rightarrow T_1$ values and observed emission maxima in nm.

Complex	Calculated $S_0 \rightarrow T_1$	Calculated $S_0 \leftarrow T_1$ adjusted[a]	Observed emission
**1**	560	593	580
**2**	560	593	588
**3**<br>**3** (90°,90°)	653<br>568	643[b]	639
**4**<br>**4** (90°,90°)	606<br>557	614[b]	600
**5**<br>**5** (90°)	589<br>553	604[b]	603
**6**<br>**6** (90°)	669<br>575	654[b]	646
**7**<br>**7** (90°)	609<br>559	617[b]	608

[a] Energy scaling factor of 0.945 applied – see reference[15].

[b] Energies averaged from two conformers.

1

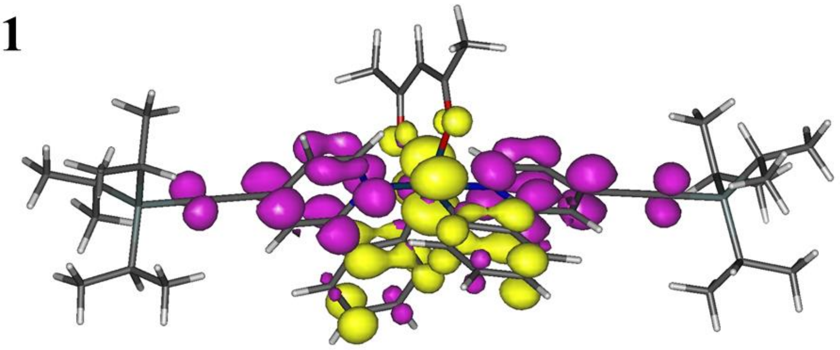

NTO	Ir	phenylene	acac	pyridyl	-C≡C-	-Si^{i}Pr$_3$
Particle	4	8	1	66	17	4
Hole	46	42	6	6	0	0

Figure S49. Natural transition orbitals (yellow = hole, purple = particle) involved in the NTOs involved in the $S_0 \leftarrow T_1$ emission for **1**. The table lists the % group contribution to each NTO. Isocontours drawn at 0.04 e bohr $^{-3/2}$.

2

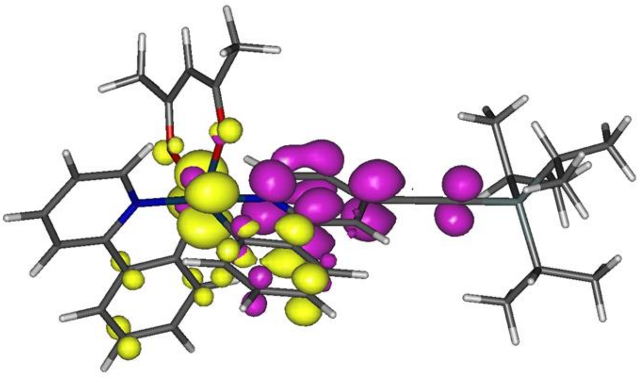

NTO	Ir	phenylene	acac	pyridyl	-C≡C-	-Si^{i}Pr$_3$
Particle	5	8	1	65	17	4
Hole	46	42	6	6	0	0

Figure S50. NTOs involved in the $S_0 \leftarrow T_1$ emission for **2**. Isocontours at 0.055 e bohr $^{-3/2}$.

3

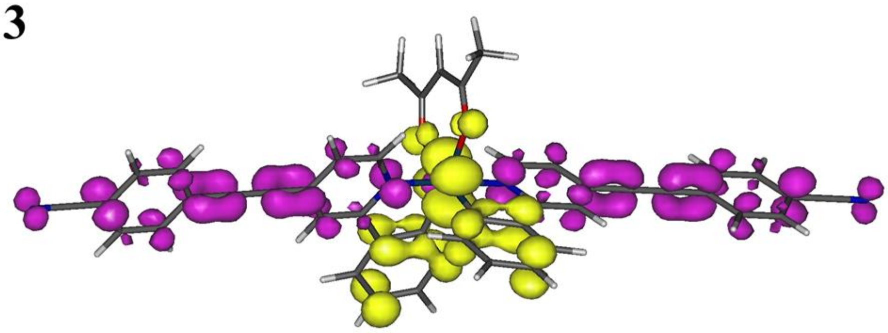

NTO	Ir	phenylene	acac	pyridyl	-C≡C-	$-C_6H_4CN$
Particle	3	3	1	34	16	43
Hole	45	43	6	6	0	0

3 (90°,90°)

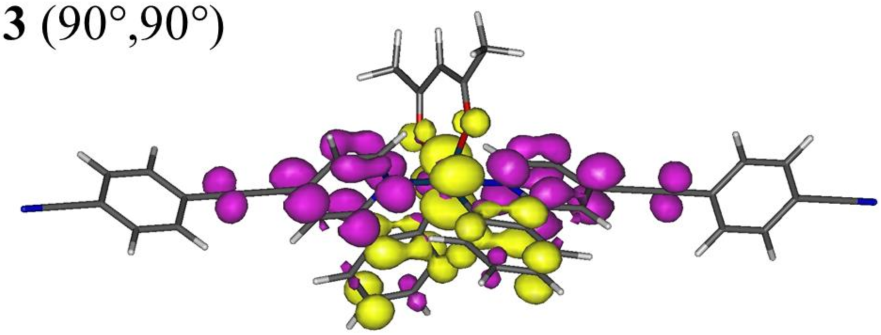

NTO	Ir	phenylene	acac	pyridyl	-C≡C-	$-C_6H_4CN$
Particle	4	8	1	66	16	5
Hole	45	43	6	6	0	0

Figure S51. NTOs involved in the $S_0 \leftarrow T_1$ emissions for the fully optimised geometry **3** where the phenylene rings attached to the ethynyl bridge are coplanar to each other and the constrained geometry **3** (90°, 90°) where the phenylene rings attached to the ethynyl bridge are constrained to be perpendicular to each other. Isocontours at 0.04 e bohr $^{-3/2}$.

4

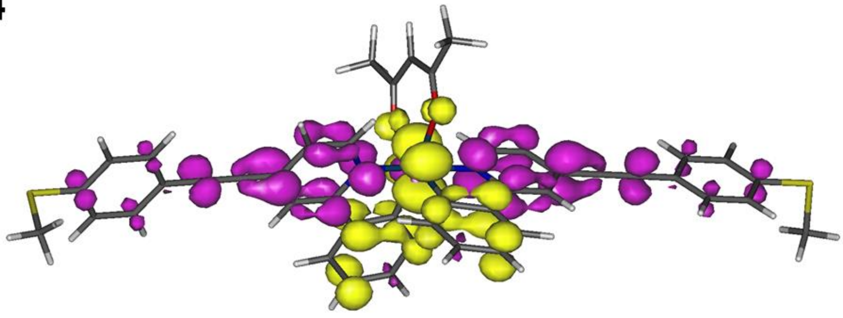

NTO	Ir	phenylene	acac	pyridyl	-C≡C-	-C_6H_4SMe
Particle	3	4	1	46	17	29
Hole	46	42	6	6	0	0

4 (90°,90°)

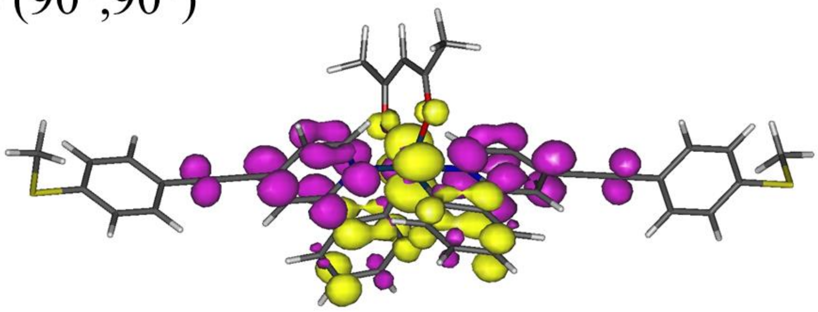

NTO	Ir	phenylene	acac	pyridyl	-C≡C-	-C_6H_4SMe
Particle	4	8	1	69	16	2
Hole	46	42	6	6	0	0

Figure S52. NTOs involved in the $S_0 \leftarrow T_1$ emissions for the fully optimised geometry **4** and the constrained geometry **4** (90°, 90°). Isocontours at 0.04 e bohr $^{-3/2}$.

5

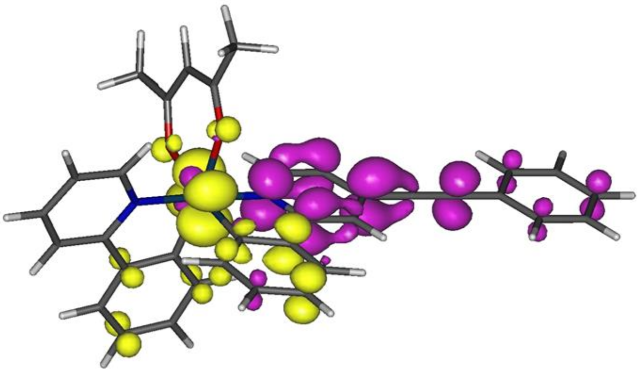

NTO	Ir	phenylene	acac	pyridyl	-C≡C-	$-C_6H_5$
Particle	4	8	1	66	16	5
Hole	45	43	6	6	0	0

5 (90°)

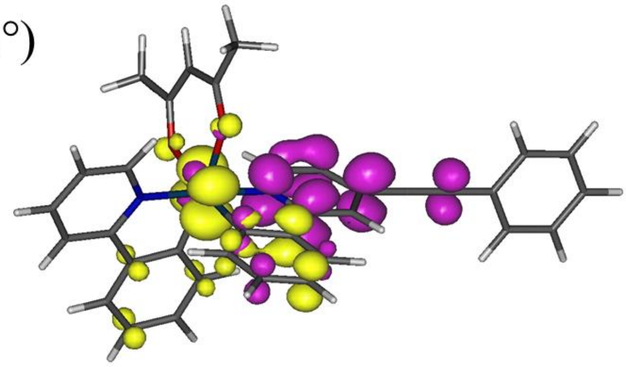

NTO	Ir	phenylene	acac	pyridyl	-C≡C-	$-C_6H_5$
Particle	5	9	1	68	15	2
Hole	45	43	6	6	0	0

Figure S53. NTOs involved in the $S_0 \leftarrow T_1$ emissions for the fully optimised geometry **5** and the constrained geometry **5** (90°). Isocontours at 0.055 e bohr $^{-3/2}$.

6

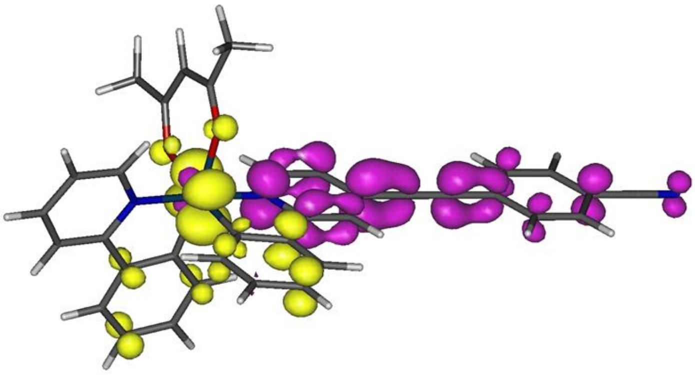

NTO	Ir	phenylene	acac	pyridyl	-C≡C-	$-C_6H_4CN$
Particle	3	3	0	33	16	45
Hole	46	42	6	6	0	0

6 (90°)

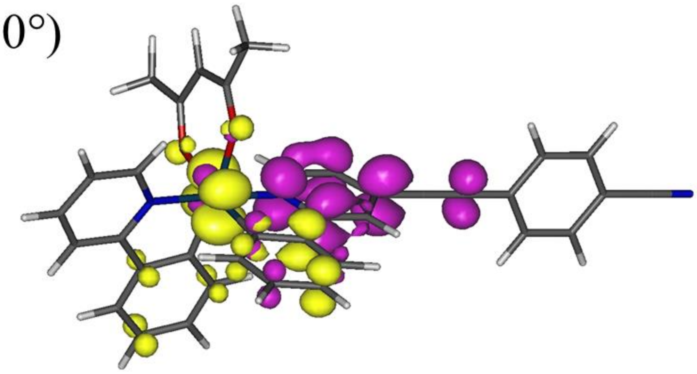

NTO	Ir	phenylene	acac	pyridyl	-C≡C-	$-C_6H_4CN$
Particle	4	8	1	68	17	2
Hole	46	42	6	6	0	0

Figure S54. NTOs involved in the $S_0 \leftarrow T_1$ emissions for the fully optimised geometry **6** and the constrained geometry **6** (90°). Isocontours at 0.055 e bohr $^{-3/2}$.

7

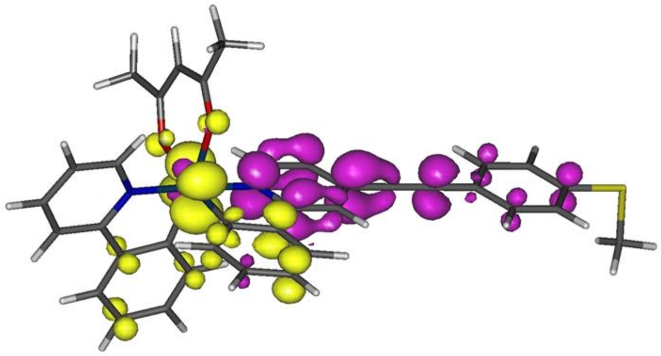

NTO	Ir	phenylene	acac	pyridyl	-C≡C-	$-C_6H_4SMe$
Particle	4	4	1	45	17	30
Hole	46	42	6	6	0	0

7 (90°)

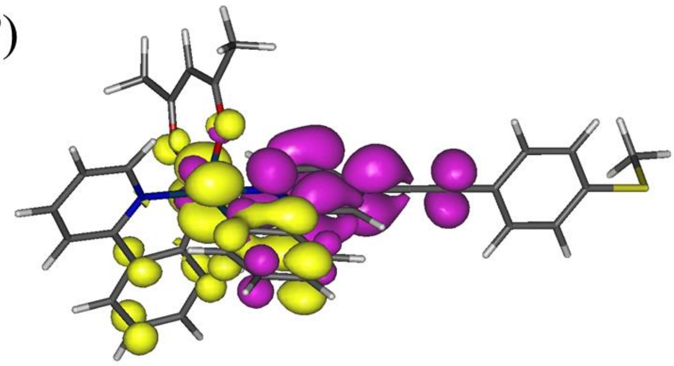

NTO	Ir	phenylene	acac	pyridyl	-C≡C-	$-C_6H_4SMe$
Particle	4	8	1	66	16	5
Hole	45	43	6	6	0	0

Figure S55. NTOs involved in the $S_0 \leftarrow T_1$ emissions for the fully optimised geometry **7** and the constrained geometry **7** (90°). Isocontours at 0.055 e bohr $^{-3/2}$.

S8. References

1. Davidson, R.; Hsu, Y.-T.; Griffiths, G. C.; Li, C.; Yufit, D.; Pal, R.; Beeby, A. Highly Linearized Twisted Iridium(III) Complexes. *Inorganic Chemistry* **2018,** 57 (22), 14450-14462 DOI: 10.1021/acs.inorgchem.8b02818.

2. Dolomanov, O. V.; Bourhis, L. J.; Gildea, R. J.; Howard, J. A. K.; Puschmann, H. OLEX2: a complete structure solution, refinement and analysis program. *Journal of Applied Crystallography* **2009,** 42, 339-341 DOI: 10.1107/s0021889808042726.

3. Sheldrick, G. A short history of SHELX. *Acta Crystallogr., Sect. A* **2008,** 64 (1), 112-122 DOI: doi:10.1107/S0108767307043930.

4. Davidson, R.; Hsu, Y.-T.; Batchelor, T.; Yufit, D.; Beeby, A. The use of organolithium reagents for the synthesis of 4-aryl-2-phenylpyridines and their corresponding iridium(iii) complexes. *Dalton Trans.* **2016,** 45 (28), 11496-11507 DOI: 10.1039/C6DT01461E.

5. Melhuish, W. H. Quantum effiencies of fluorescence of organic substances: effect of solvent and concentration of the fluorescent solute. *J. Phys. Chem.* **1961,** 65 (2), 229-235 DOI: 10.1021/j100820a009.

6. Karstens, T.; Kobs, K. Rhodamine B and rhodamine 101 as reference substances for fluorescence quantum yield measurements. *The Journal of Physical Chemistry* **1980,** 84 (14), 1871-1872 DOI: 10.1021/j100451a030.

7. M. J. Frisch; G. W. Trucks; H. B. Schlegel; G. E. Scuseria; M. A. Robb; J. R. Cheeseman; G. Scalmani; V. Barone; B. Mennucci; G. A. Petersson; H. Nakatsuji; M. Caricato; X. Li; H. P. Hratchian; A. F. Izmaylov; J. Bloino; G. Zheng; J. L. Sonnenberg; M. Hada; M. Ehara; K. Toyota; R. Fukuda; J. Hasegawa; M. Ishida; T. Nakajima; Y. Honda; O. Kitao; H. Nakai; T. Vreven; J. A. Montgomery, J., J. E. Peralta, F. Ogliaro, M. Bearpark, J. J. Heyd, E. Brothers, K. N. Kudin, V. N. Staroverov, R. Kobayashi, J. Normand, K. Raghavachari, A.

Rendell, J. C. Burant, S. S. Iyengar, J. Tomasi, M. Cossi, N. Rega, J. M. Millam, M. Klene, J. E. Knox, J. B. Cross, V. Bakken, C. Adamo, J. Jaramillo, R. Gomperts, R. E. Stratmann, O. Yazyev, A. J. Austin, R. Cammi, C. Pomelli, J. W. Ochterski, R. L. Martin, K. Morokuma, V. G. Zakrzewski, G. A. Voth, P. Salvador, J. J. Dannenberg, S. Dapprich, A. D. Daniels, Ö. Farkas, J. B. Foresman, J. V. Ortiz, J. Cioslowski, and D. J. Fox. *Gaussian 09*, A.1; Gaussian, Inc: Wallingford CT, 2009.

8. Lee, C.; Yang, W.; Parr, R. G. Development of the Colle-Salvetti correlation-energy formula into a functional of the electron density. *Physical Review B* **1988,** 37 (2), 785-789 DOI: 10.1103/PhysRevB.37.785.

9. Hay, P. J.; Wadt, W. R. Ab initio effective core potentials for molecular calculations. Potentials for K to Au including the outermost core orbitals. *The Journal of Chemical Physics* **1985,** 82 (1), 299-310 DOI: 10.1063/1.448975.

10. Hay, P. J.; Wadt, W. R. Ab initio effective core potentials for molecular calculations. Potentials for the transition metal atoms Sc to Hg. *The Journal of Chemical Physics* **1985,** 82 (1), 270-283 DOI: 10.1063/1.448799.

11. Petersson, G. A.; Al-Laham, M. A. A complete basis set model chemistry. II. Open-shell systems and the total energies of the first-row atoms. *The Journal of Chemical Physics* **1991,** 94 (9), 6081-6090 DOI: 10.1063/1.460447.

12. Petersson, G. A.; Bennett, A.; Tensfeldt, T. G.; Al-Laham, M. A.; Shirley, W. A.; Mantzaris, J. A complete basis set model chemistry. I. The total energies of closed-shell atoms and hydrides of the first-row elements. *The Journal of Chemical Physics* **1988,** 89 (4), 2193-2218 DOI: 10.1063/1.455064.

13. Allouche, A.-R. Gabedit—A graphical user interface for computational chemistry softwares. *Journal of Computational Chemistry* **2011,** 32 (1), 174-182 DOI: https://doi.org/10.1002/jcc.21600.

14. O'Boyle, N. M.; Tenderholt, A. L.; Langner, K. M. cclib: A library for package-independent computational chemistry algorithms. *Journal of Computational Chemistry* **2008,** 29 (5), 839-845 DOI: https://doi.org/10.1002/jcc.20823.

15. Benjamin, H.; Fox, M. A.; Batsanov, A. S.; Al-Attar, H. A.; Li, C.; Ren, Z.; Monkman, A. P.; Bryce, M. R. Pyridylpyrazole N^N ligands combined with sulfonyl-functionalised cyclometalating ligands for blue-emitting iridium(III) complexes and solution-processable PhOLEDs. *Dalton Transactions* **2017,** 46 (33), 10996-11007 DOI: 10.1039/C7DT02289A.
